# Synthesis, characterization, and investigation of photochemical and in vitro properties of novel Zn(II) phthalocyanine

**DOI:** 10.55730/1300-0527.3699

**Published:** 2024-10-25

**Authors:** Yasemin BAYĞU YILDIZ, Nilgün KABAY, Burak YILDIZ, İpek ÖMEROĞLU, Mahmut DURMUŞ, E. Rıza KARAGÜR, Hakan AKÇA, Çağrı ERGİN, Yaşar GÖK

**Affiliations:** 1 Tavas Vocational High School, Pamukkale University, Denizli, Turkiye; 2 Department of Biomedical Engineering, Faculty of Technology, Pamukkale University, Denizli, Turkiye; 3 Aliağa Vocational High School, Ege University, İzmir, Turkiye; 4 Department of Chemistry, Faculty of Science, Gebze Technical University, Kocaeli, Turkiye; 5 Department of Medical Genetic, School of Medicine, Pamukkale University, Denizli, Turkiye; 6 Department of Medical Microbiology, School of Medicine, Pamukkale University, Denizli, Turkiye; 7 Department of Chemical Engineering, Faculty of Engineering and Natural Sciences, Uşak University, Uşak, Turkiye

**Keywords:** Photodynamic therapy (PDT), singlet oxygen, in vitro investigation, click reaction, zinc phthalocyanine

## Abstract

A new nonperipheral zinc(II) phthalocyanine bearing octa carboxylic acid ethyl ester derivative substituted triazole attached propylmercaptothiobenzylmercapto derivative was synthesized via the tetramerization reaction of phthalonitrile. The photochemical in vitro photodynamic activity of zinc(II) phthalocyanine (**ZnPc-I**), such as human nonsmall cell lung carcinoma cell lines, was investigated in this study. The singlet oxygen generation property of novel zinc(II) phthalocyanine (**ZnPc-I**) was also examined due to the significantly high singlet oxygen quantum yield of **ZnPc-I** (F_D_ = 0.66). The antiproliferative effects of **ZnPc-I** were also investigated on the A549 and H1299 cell lines, and the results demonstrated that **ZnPc-I** had a strong antiproliferative effect on both cell lines.

## 1. Introduction

Phthalocyanines and their derivatives are one of the many important substances for a number of applications, such as dyes and pigments [1,2], electrochromic [3–5], gas sensors [6–8], solar cells [9–12], and photodynamic therapy (PDT) in medical applications for cancer therapy [13–15]. The low solubility of phthalocyanines and metal complexes limited their various applications. Tailoring groups such as ethyl ester-linked triazole containing nonperipheral substitutions of the phthalocyanine core are one way of the solubilities of phthalocyanines [16–18]. Phthalocyanine zinc(II) complexes are especially important as photosensitizers with short triplet lifetimes. In addition to that, the diamagnetic characters of zinc(II) fabricate high singlet oxygen owing to their high triplet quantum yields and long triplet time phthalocyanine [19–21].

The importance of PDT, which consists of three main components such as light source, oxygen, and photosensitizer, is increasing day by day [22]. The use of light and some chemicals as a photosensitizer has been successful in some diseases such as psoriasis and in some types of cancer [23–25]. PDT has a selective and local destructive effect on diseased tissues and cells. This property relies on the special character of photosensitizers to preferentially gather in the diseased tissue and generate produced singlet oxygen to death target cells or tissues. Metallophthalocyanines are great candidates for use as photosensitizers because of their strong and long-wavelength absorption in the red/near-infrared region, where tissue penetration is high [26].

The type of metal ion in the central cavity can be changed, or substituents can be added at the axial, peripheral, or nonperipheral positions, to alter the physical and chemical properties of phthalocyanines [27]. Because phthalocyanine compounds contain four benzene rings, some of the main problems with them include reduced solubility and aggregation in solutions. Such situations can be avoided by substituting functional groups on the benzene rings in the peripheral and nonperipheral, such as the tetra and octa positions [28]. In comparison with substitution at the peripheral positions, substitution at the nonperipheral positions redshifts the Q-band significantly. Moreover, the Q-band will shift to even longer wavelengths in the presence of electron-donating sulfur groups in addition to replacement at nonperipheral locations [29].

The nonperipheral-carboxylate derivative of Zn(II) phthalocyanine (**ZnP-I)** was synthesized in this study. The chemical structure of the synthesized compound was characterized using spectroscopic techniques such as elemental analysis, hydrogen-1 nuclear magnetic resonance (^1^H NMR), carbon-13 NMR (^13^C NMR), Fourier-transform infrared spectroscopy (FTIR), ultraviolet visible (UV-vis) spectroscopy, and mass spectrometry (MS). The novel zinc(II) phthalocyanine was studied to determine the singlet oxygen quantum yield and in vitro antitumor efficiency on lung cancer cell lines A549 (CCl-185) and H1299 (CRL-5803).

## 2. Experimental section

The materials and equipment, photochemical parameter, and in vitro studies are given in the Supplementary Information.

### 2.1. Synthesis

#### 2.1.1. Preparation of the 4-pent-4-ylsuphonyl benzoic acid (3)

4-Mercapto benzoic acid methyl ester (1) (1.18 g, 7.02 mmol), 5-chloro-1-pentyn (**2**) (0.72 g, 7.02 mmol), NaI (1.05 g, 7.02 mmol), and an excess amount of anhydrous K_2_CO_3_ (9.68 g, 70.2 mmol were added to a two-necked flask and dissolved 50 mL of dry acetone. Then, the reaction mixture was refluxed and stirred under an argon atmosphere at reflux temperature for 24 h. After the reaction was completed, the mixture was cooled to room temperature. The reaction mixture was filtered off and washed with acetone and then the solvent was evaporated to dryness under reduced pressure. The resulting yellow crude product was purified by column chromatography on silica gel using dichloromethane as eluent to obtain a waxy pale yellow product. Yield: 1.04 g (63.3%). ^1^H NMR (400 MHz, CDCl_3_): δ 7.92 (d, 2H, Ar–H), 7.31 (d, 2H, Ar–H), 3.88 (s, 3H, −CH_3_), 3.10 (t, 2H, SCH_2_), 2.35 (q, 2H, −CH_2_), 2.00 (s, 1H, C≡CH), 1.88 (t, 2H, CH_2_). ^13^C NMR (100 MHz, CDCl_3_): δ 166.99 (C=O), 143.75, 130.19, 127.08, 126.81, 83.17, 69.65 (−C≡CH), 52.29 (−OCH_3_), 30.98 (−SCH_2_), 27.66, 17.76 (CH_2_). FTIR (ATR, cm^−1^): 3294 (−C≡CH), 2940–2844 (CH)_alip_., 2117 (C≡C), 1713 (C=O). MS (m/z): 233.565 [M–H]^−^. Anal. cald. for C_13_H_14_O_2_S: C, 66.66; H, 5.98. Found: C, 66.84; H, 6.17.

#### 2.1.2. Preparation of (4-pent-4-ylsulphonyl phenyl)methanol (4)

4-Pent-4-ylsulphonyl benzoic acid methyl ester (3) (1.88 g, 8.03 mmol) and NaBH_4_ (1.85 g, 48.61 mmol) were added to a two-necked flask and dissolved in 30 mL of dry tetrahydrofuran (THF). The reaction mixture was stirred under an argon atmosphere at 65 °C for 15 min. After that, dry methanol (30 mL) was added dropwise to the reaction mixture and then refluxed for 4 h. The completion of the reaction was checked by thin layer chromatography (TLC) [silica gel (chloroform)]. After the reaction was completed, the mixture was cooled to room temperature and concentrated aqueous NH_4_Cl (60 mL) solution was added to this solution and stirred for 90 min at room temperature. The product was extracted with diethyl ether (3 × 40 mL) and washed with concentrated brine and then dried over anhydrous MgSO_4_. The reaction mixture was filtered off and then the solvent was removed using a rotatory evaporator. Purification of the product was accomplished by column chromatography on silica gel with chloroform as an eluent to yield a colorless waxy product. Yield: 1.38 g (83.43%). ^1^H NMR (400 MHz, CDCl_3_): δ 7.31 (d, 2H, Ar–H), 7.24 (d, 2H, Ar–H), 4.61 (s, 2H, −OCH_2_), 3.02 (t, 2H, −SCH_2_), 2.33 (q, 2H, −CH_2_), 2.20 (s, 1H, −OH), 1.98 (s, 1H, C≡CH), 1.83 (t, 2H, −CH_2_).^13^C NMR (100 MHz, CDCl_3_): δ 139.00, 135.55, 129.69, 127.83, 83.51, 69.42 (−C≡C), 65.04 (−OCH_2_), 32.68 (−SCH_2_), 27.93, 17.66 (−CH_2_). FTIR (ATR, cm^−1^): 3378 (O–H), 3290 (C≡CH), 3074, 3019, 2938, 2869 (C–H) _aliph_. 2116 (C≡C). MS (m/z): 205.118 [M–H]^−^. Anal. cald. for C_12_H_14_O_2_S: C, 69.90; H, 6.76. Found: C, 70.19; H, 6.43.

#### 2.1.3. Preparation of the {4-[3-(4-hydroxymethylphenyl)propyl]-[1,2,3]triazol-1-yl}acetic acid ethylester (6)

An orange aqueous suspension (35 mL) of sodium-L-ascorbate (0.5 g, 2.56 mmol) and Cu(CH_3_COO)_2_. H_2_O (0.25 g, 1.24 mmol) was added to a mixture of 4-pent-4-ylsulphonyl phenyl)methanol (4) (1.32 g, 6.4 mmol) and 2-azido ethyl acetate (**5**) (1.65 g, 12.81 mmol) in *tert*-butanol (35 mL) and stirred at room temperature overnight. After that, the reaction mixture was poured into water (70 mL) and extracted with chloroform (3 × 50 mL). The collected organic phase was washed with concentrated brine solution (50 mL) and dried over anhydrous MgSO_4_. The crude product was filtered off, washed with chloroform, and evaporated under reduced pressure to dryness. The waxy colorless product was stirred with diethyl ether to solidify and filtered and then dried over P_4_O_10_. Yield: 1.8 g (83.96%), m.p: 70–72 °C. ^1^H NMR (400 MHz, CDCl_3_): δ 7.45 (s, 1H, HC=C), 7.31 (m, 4H, Ar–H), 5.12 (s, 2H, −NCH_2_), 4.65 (s, 2H, −CH_2_OH), 4.27 (m, 2H, OCH_2_), 2.97 (t, 2H, −SCH_2_), 2.88 (m, 4H, CH_2_), 2.04 (s, 1H, OH), 1.30 (t, 3H, CH_3_). ^13^C NMR (100 MHz, CDCl_3_): δ 166.68 (C=O), 139.05, 129.76, 127.91, 65.09 (ArCH_2_O), 62.65 (OCH_2_), 51.07 (C–N), 33.19 (SCH_2_), 28.75, 24.58 (CH_2_), 14.32 (CH_3_). FTIR (ATR, cm^−1^): 3341 (O–H), 3121 (triazole), 3063, 2950–2841 (C–H) aliph. 1746 (C=O). MS (m/z): 205.118 [M–H]^−^. Anal. cald. for C_16_H_21_N_3_O_3_S: C, 57.31; H, 6.26; N, 12.53. Found: C, 57.05; H, 6.02; N, 12.78.

#### 2.1.4. Preparation of the {4-[3-(4-iodomethylphenyl)propyl]-[1,2,3]triazol-1-yl}acetic acid ethylester (7)

Trimethylbromosilane (2.47 g, 16.12 mmol) was added to a solution of compound **6** (1.8 g, 5.37 mmol) and NaI (2.42 g, 16.12 mmol) in dry acetonitrile (100 mL) under an argon atmosphere at room temperature. The reaction mixture was stirred at the same temperature for 7 min and then poured into dichloromethane (100 mL) and water (100 mL), respectively. The organic phase was separated and washed with water (2 × 100 mL) and concentrated aqueous sodium thiosulfate solution (75 mL) and then dried over anhydrous MgSO_4_. Yield: 2.12 g (83.14%), m.p: 89 °C. ^1^H NMR (400 MHz, CDCl_3_): δ 7.42 (s, 1H, HC=C), 7.23 (d, 2H, C–H)_arom_, 7.20 (s, 2H, C–H)_arom_.,5.12 (s, 2H, NCH_2_), 4.44 (s, 2H, ICH_2_), 4.27 (m, 2H, −OCH_2_), 2.99 (s, 2H, CH_2_), 2.89 (t, 2H, −SCH_2_), 2.06 (m, 2H, −CH_2_−), 1.30 (t, 3H, CH_3_). ^13^C NMR (100 MHz, CDCl_3_): δ 166.67 (C=O), 147.50 (C=CH)_arom_., 139.94, 136.71, 129.48, 129.12, 122.65, 62.62 (−OCH_2_), 51.03 (−NCH_2_), 32.61 (−SCH_2_), 28.69, 24.60 (−CH_2_-)_aliph_., 14.34(−CH_3_), 5.97 (ICH_2_). FTIR (ATR, cm^−1^): 3121 (triazole), 3064, 2996–2950 (C–H)_aliph_. 1746 (C=O). MS (m/z): 467.01 [M+Na–H]^−^, 468.13 [M+Na]^+^. Anal. cald. for C_16_H_20_N_3_O_3_SI: C, 43.14; H, 4.49; N, 9.43. Found: C, 43.69; H, 4.77; N, 9.72.

#### 2.1.5. Preparation of the (4-{3-[4-(2,3-dicyano-4-{4-[3-(1-ethoxycarbonylmethyl-1H-[1,2,3]triazol-4-yl)-propylsulfonyl}-phenylsulfonylmethyl)-phenylsulfonyl]propyl}-[1,2,3]triazol-1-yl)aceticacid ethylester][1,2,3]triazol-1-yl} acetic acid ethylester (9)

3,6-Dimercapto phthalonitrile (8) (0.41 g, 2.17 mmol) and anhydrous K_2_CO_3_ (0.90 g, 6.51 mmol) were mixed in dry acetone (50 mL) under an argon atmosphere at room temperature for 35 min. Then, the solution of compound (**7**) (2.12 g, 4.76 mmol) in dry acetone and dichloromethane (20: 10 mL) was added to this mixture under the same conditions. The reaction mixture was stirred overnight and then evaporated to dryness under reduced pressure. Water and chloroform [150 mL (1:1)] were added to the residue and the organic phase was separated and then dried over anhydrous MgSO_4_. The crude product was purified by column chromatography [silica gel (dichloromethane: methanol) (95:5)]. Yield: 0.25 g (16.84%), m.p: 132 °C. ^1^H NMR (400 MHz, CDCl_3_): δ 7.69 (d, 2H, CH=C)., 7.49 (s, 2H, C–H)_arom_., 7.42 (s, 4H, C–H)_arom._ 7.25 (s, 4H, C–H)_arom_., 5.13 (s, 4H, −NCH_2_), 4.23 (s, 4H, ArCH_2_S−), 3.86 (s, 4H, −SCH_2_), 2.98 (t, 4H, −CH_2_−), 2.88 (m, 4H, −CH_2_−), 2.06 m (4H, −CH_2_), 1.57 (s, 6H, CH_3_). ^13^C NMR (100 MHz, CDCl_3_): δ 166.87 (C=O), 147.37, 145.47 (CH=C)_arom._, 140.48, 136.77, 131.88, 131.32, 129.30, 129.19, 123.74, 122.33 (C=C), 113.90 (C≡N), 53.01 (C–N), 50.62, 37.61, 32.50 (−SCH_2_−), 28.49 (-CH_2_-), 24.41 (CH_3_). FTIR (ATR, cm^−1^): 3126 (triazole), 3071 (Ar–H), 2982, 2935 (C–H)_aliph_. 2223 (C≡N), 1742 (C=O). MS (m/z): 826.453 [M]^+^. Anal. cald. for C_40_H_42_N_8_O_4_S_4_: C, 58.11; H, 5.08; N, 13.55. Found: C, 58.40; H, 5.39; N, 13.19.

#### 2.1.6. Preparation of the ZnPc-I

Compound **9** (0.249 g, 0.3 mmol) and anhydrous Zn(CH_3_COO)_2_ (0.0201 g, 0.11 mmol) were solved in 3 mL of dry n-pentanol. Then, 5 drops of diazabicyclo[5.4.0]undec-7-ene (DBU) was added to the reaction mixture. The mixture was stirred at 155 °C for 24 h under an argon atmosphere and observed by TLC [silica gel (dichloromethane: methanol) (95:5)]. After the reaction was completed, the reaction was cooled to room temperature. The crude product was filtered off and then washed with water, methanol and diethyl ether, respectively. The crude product was purified by column chromatography [silica gel (dichloromethane: methanol) (95:5)] to give a dark brown crystalline solid. Yield: 0.09 g (35.62%), m.p > 300 °C. ^1^H NMR (400 MHz, DMSO–d_6_): δ 7.83 (s, 8H, CH=C), 7.46 (br, 8H, C–H)_arom._, 7.35 (br, 16H, C–H)_arom.,_ 7.20 (br, 24H, C–H)_arom._, 5.29 (s, 18H, N–CH_2_), 4.02 (br, 18H, Ar–CH_2_S−), 3.36 (s, 18H, −SCH_2_), 2.88 (br, 8H, −CH_2_−), 2.72 (br, 10H, −CH_2_−), 2.63 (br, 8H, −CH_2_−), 1.85 (m, 8H, −CH_2_−), 1.48 (s, 24H, −CH_3_). ^13^C NMR (100 MHz, CDCl_3_): δ 167.75 (C=O), 146.42 (C=CH)_arom._, 135.27, 130.07, 128.37, 123.92, 123.44, 50.69 (C-N), 31.79, 28.88 (−SCH_2_), 28.03. FTIR (ATR, cm^−1^): 3141 (triazole), 3074 (Ar–H), 2928, 2861 (C–H)_aliph_. 1743 (C=O), 1621 (C=N). UV-vis (DMSO): λ_max_, nm (log ɛ): 789 (4.75), 713 (4.16), 360 (4.44). MS (m/z): 3279 [M–2(OCH_2_CH_3_)]^−^, 3501 [M+2K+3H_2_O]^+^. Anal. cald. for C_160_H_168_N_32_O_16_S_16_Zn: C, 56.96; H, 5.02; N, 13.29; Zn, 1.94. Found: C, 57.34; H, 4.61; N, 12.83.

#### 2.1.7. Cell culture and viability

The A549 and H1299 cells were cultured in cell culture dishes at 37 °C under a humidified atmosphere containing 5% CO_2_ air in a completed medium (DMEM and RPMI 1640 supplemented with 10% fetal bovine serum and 1% penicillin-streptomycin, respectively).

3-(4,5-dimethylthiazol-2-yl)-2,5-diphenyltetrazolium bromide (MTT) was used to measure cell viability in order to assess the **ZnPC-I** effect.

The **ZnPc-I** was dissolved in medium with 10% DMSO to prepare for the proliferation experiment. First, the master stock was set as 2000 μg/mL and the **ZnPc-I** was treated as final concentrations of 1, 10, 50, 100, 250, 500, and 1000 μg/mL.

After the A549 and H1299 cell lines reached 80% confluence, the cells were trypsinized, counted using a hemocytometer 3 times, and seeded at 2 × 10^3^ cells per well in 96-well tissue culture plates. The medium was removed after 24 h, and the cells were incubated for 72 h in fresh medium that contained **ZnPc-I** at various concentrations (1, 10, 50, 100, 250, 500, and 1000 μg/mL). At the end of the incubation periods, the cell viability in each group was measured using the Cell Proliferation Kit I (MTT) according to the manufacturer’s instructions (Sigma-Aldrich Chemical Co., St. Louis, MO, USA). Formazan formation was quantified spectrophotometrically at 560 nm using a microplate reader.

## 3.Results and discussion

### 3.1.Synthesis and characterization

The novel compounds (**3**, **4**, **6**, **7**, and **9**) and the zinc(II) phthalocyanine (**ZnPc-I**) were synthesized and then characterized by known standard analytical and spectroscopic methods. The synthesis steps of the new compounds are shown in the Scheme. The target compound (**ZnPc-I**) was obtained in six steps; first, 4-mercapto benzoic acid methyl ester (1) and 5-chloro-1-pentyn (2) was refluxed in the presence of anhydrous K_2_CO_3_ to prepare compound **3**. The disappearance of the −SH chemical shift and characteristic resonances of the precursor compounds (**1** and **2**) indicated the formation of compound **3**. The reduction reaction of compound **3** was carried out under reflux temperature in dry THF/methanol using NaBH_4_ to obtain compound **4** at a high yield (83.43%). The important variety of the ^1^H NMR and FTIR spectra of compounds **3** and **4** was the conversion of the carbonyl group to CH_2_ moiety (Figures S1–S8). 1,3-Dipolar cycloaddition reaction [16–18] was performed between compound **4** and 2-azido ethyl acetate (5) in the presence of copper(II) acetate monohydrate in the presence of sodium-L-ascorbate in water/tert-butanol mixture (1:1) at room temperature to obtain compound **6** at a high yield (83.96%). The characteristic triazole resonance at δ = 7.45 ppm in the ^1^H NMR and carboxy group chemical shifts in the ^13^C NMR spectra at δ = 166.68 ppm confirmed the formation of the addition reaction (Figures S9–S11). The conversion of compound **6** to the iodo derivative 7 was prepared at a high yield (83.14%) when one equivalent of compound **6** was reacted with three equivalents of trimethylbromo silane and sodium iodide at room temperature. According to the spectral data, the transformation of the −CH_2_–OH moiety to the −CH_2_–I group was achieved. The disappearance of characteristic resonances belonging to the −CH_2_OH moiety and the appearance of a new −CH_2_–I group in the FTIR, NMR, and MS spectra for compound **6** at 3341 cm^−1^, δ = 4.65, 65.09 ppm m/z = 205.118 [M–H]^−^, respectively, and appearance of novel resonances δ = 4.44 and 5.97 ppm in the ^1^H and ^13^C NMR and m/z = 468.13 [M+Na]^+^ data also indicated the formation of compound **7** (Figures S12–S15). Compound **9** was prepared between compound **7** and 3,6-dimercapto phthalonitrile (8) in K_2_CO_3_ and dry acetone at room temperature overnight at a low yield of 16.84%. In the FTIR spectrum of compound **9**, the disappearance of the peak belonging to the −SH groups supported the formation of the structure (Figure S16). The ^1^H NMR spectrum of compound **9** confirmed the formation of the 3,6-disubstituted phthalonitrile derivative with the presence of triazole at δ = 7.69 ppm and other chemical shifts in the ester groups at δ = 1.57–2.06 ppm (Figure S17). The disappearance of signals related to the −SH protons supported the formation of the desired addition reaction. The peaks at δ = 166.87, 145.47, and 113.90 ppm in the ^13^C NMR spectrum of this compound can be attributed to the C=O, triazole carbon, and nitrile moieties, respectively (Figure S18). In addition to that, the formation of the 3,6-disubstituted dinitrile compound (**9**) was also confirmed by MS spectral data at m/z = 826.453 [M]^+^ (Figure S19).

The reaction of compound **9** in dry n-pentanol under an argon atmosphere containing a few drops of DBU with anhydrous Zn(CH_3_COO)_2_ gave the desired metallo-phthalocyanine (**ZnPc-I**) at an acceptable yield (35.62%). The formation of novel **ZnPc-I** was determined by spectral and analytical techniques. In the ^1^H NMR spectrum of **ZnPc-I**, triazole, Pc, and other aromatic protons were observed at δ = 7.83, 7.46, and 7.35–7.20 ppm, respectively (Figure S20). The chemical shifts in the ^13^C NMR spectrum of the same compound, and the deformation of C≡N resonances in the precursor dinitrile compound (**9**) indicated the macrocyclization reaction. In addition to that, carbon resonances at δ = 167.75 and 146.42 ppm related to the C=O and triazole moieties could be attributed to the formation of the phthalocyanine compound (**ZnPc-I**) (Figure S21). The absence of nitrile and presence of C=N stretching vibrations at 2223 and 1621 cm^−1^, in the FTIR spectrum of this compound, respectively, also indicated the phthalocyanine formation (Figure S22). The mass spectrum of this compound at m/z = 3279.085 [M–2(OC_2_H_5_)]^−^ and 3501.024 [M+2K+3H_2_O]^+^ supported the proposed zinc phthalocyanine formation (Figure S23).

### 3.2. Ground state electronic absorption property of ZnPc-I

Two major absorption bands, referred to as the Q band at 650–700 nm and Soret (B band) band at 300–350 nm, were monitored in the ground state electronic absorption spectra of the phthalocyanine compounds [30]. The Q band of novel **ZnPc-I** was observed at 789 nm in DMSO and is given in the Table. The newly synthesized zinc phthalocyanine (**ZnPc-I**) displayed the monomeric and nonaggregated behaviors with narrow and single Q band [31] (Figure S24). The nonperipheral substituted **ZnPc-I** showed red-shifted at the Q band absorption, as expected, due to the α position of sulfur linked substituents [32,33]. This compound did not show any fluorescence properties in DMSO.

**Figure 1:**
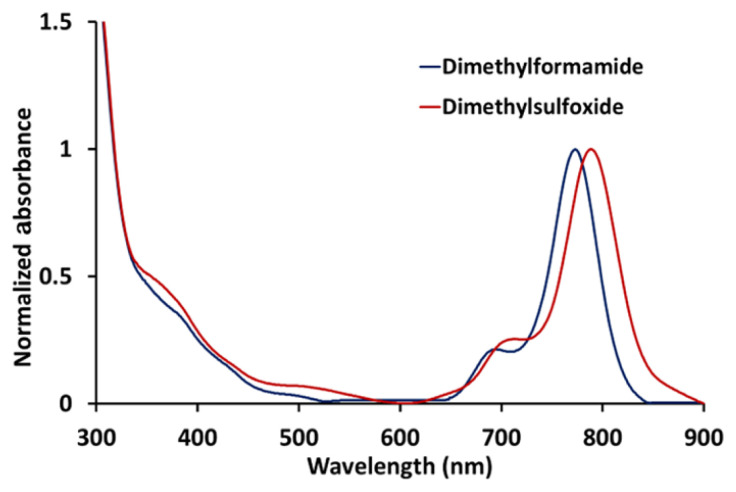
Normalized absorption spectra of **ZnPc-I** in DMSO and DMF.

**Figure 2:**
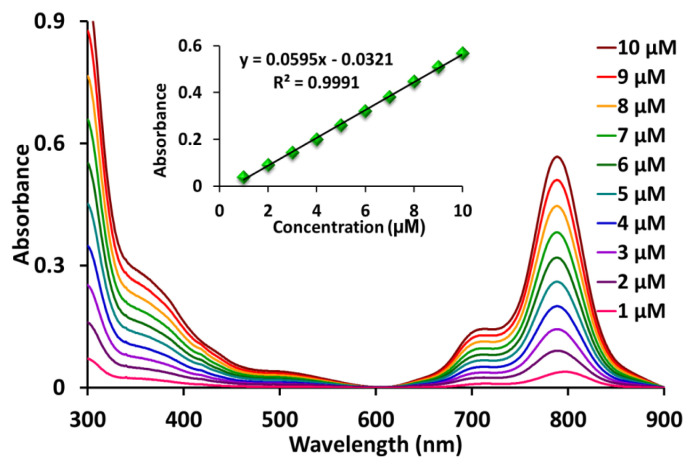
Absorption spectra of **ZnPc-I** (from 1 to 10 μM in DMSO) at different concentrations. Inset: absorbance vs. concentration at maximum.

It is very important to define an appropriate solvent for examination of the photophysicochemical measurements of phthalocyanine compounds. Therefore, the aggregation behavior of the newly synthesized zinc phthalocyanine (**ZnPc-I**) was investigated in DMF and DMSO (Figure 1). In addition, the aggregation attitude of this compound at different concentrations was studied and it did not indicate any aggregation from 1 to 10 μM (Figure 2).

### 3.3. Singlet oxygen quantum yields (ΦΔ)

**Figure 3:**
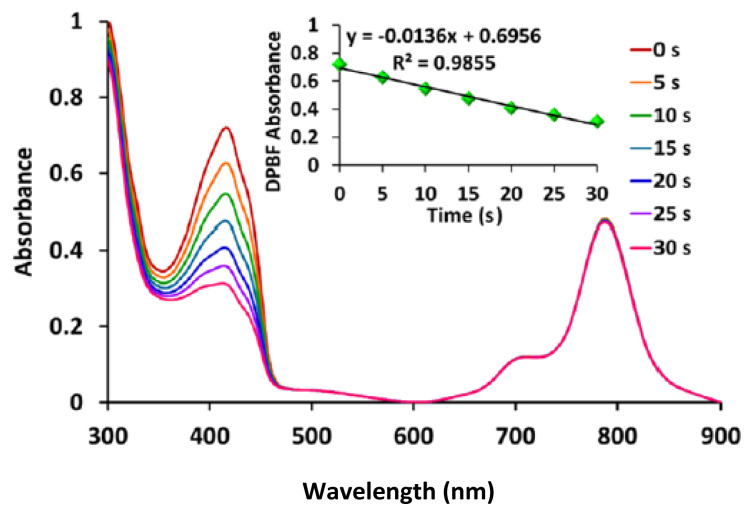
Absorption changes during the definition of singlet oxygen quantum yields for **ZnPc-I** in DMSO using DPBF as a singlet oxygen quencher.

One of the most significant factors in PDT applications is the singlet oxygen produced by photosensitizing compounds. The presence of diamagnetic metal atom in the phthalocyanine core can increase the singlet oxygen quantum yield (Φ_Δ_) [35]. As a result of the measurements, it was determined that **ZnPc-I** (Φ_Δ_ = 0.66) showed almost the same singlet oxygen quantum yield as unsubstituted ZnPc (Φ_Δ_= 0.67) in DMSO. The singlet oxygen quantum value of the new zinc(II) phthalocyanine is given in Table. According to this result, the newly synthesized phthalocyanine compound was seen as a very convenient photosensitizer in PDT applications (Figure 3). The synthesized **ZnPc-I** showed a higher singlet oxygen quantum yield than peripheral (Φ_Δ_ = 0.36) and nonperipheral (Φ_Δ_ = 0.60) octa-substituted ZnPcs with polyether linked triazole moieties carboxylic groups [36]. Furthermore, **ZnPc-I** indicated higher singlet oxygen quantum yield than octa-substituted ZnPc at the peripheral positions with a 2-mercaptopyridine groups (Φ_Δ_ = 0.51) [37] and peripheral octa-substituted ZnPc with mercaptopropionic acid groups (Φ_Δ_= 0.27) [38].

### 3.4. Antiproliferative effect of ZnPc-I on the A549 and H1299 cells

The potential antiproliferative effects of the **ZnPc-I** compound on A549 and H1299 cells were also examined. Therefore, the A549 and H1299 cells were treated with different concentrations of ZnPc compound s. After 72 h of treatment, the MTT method was used for determination of the antiproliferative effects of the ZnPc compound.

**Figure 4:**
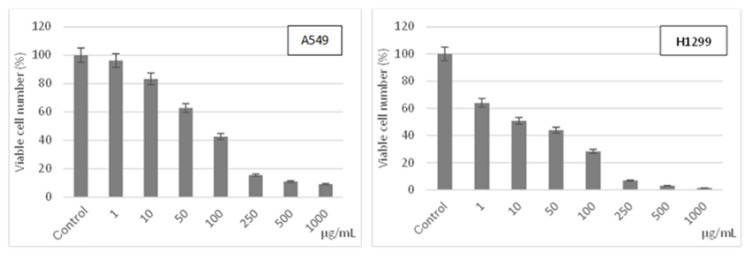
Antiproliferative effect of compound **ZnP-I** on the A549 and H1299 cells.

It is clearly shown in Figure 4 that the **ZnPc-I** compound had an antiproliferative effect on both the A549 and H1299 cell lines. This effect started even with the lowest concentration (1 μg/mL) and continued to increase in a dose-dependent manner. Moreover, Figure 4 also shows that this antiproliferative effect of the **ZnPc-I** compound was stronger on the H1299 (half-maximal inhibitory concentration (IC_50_): 10 ± 0.95 ng/mL) cells when compared with the A549 (IC_50_: 58.4 ± 7.16 ng/mL) cells.

## 4. Conclusions

Nonperipheral zinc(II) phthalocyanine, including the octa carboxylic acid ethyl ester derivative (**ZnPc-I**) of this compound substituted triazole attached propyl-mercapto thiobenzylmercapto derivative, was synthesized within the scope of this study. The structure of the novel zinc(II) phthalocyanine was characterized with spectroscopic techniques such as FTIR, NMR (^1^H and ^13^C), UV-vis, matrix-assisted laser desorption/ionization time-of-flight (MALDI-TOF) mass spectra, and elemental analysis. It was determined that the singlet oxygen quantum yield of the zinc(II) phthalocyanine (**ZnPc-I**, Φ_Δ_ = 0.66) was quite similar to that of unsubstituted ZnPc (Φ_Δ_ = 0.67). This showed that zinc(II) phthalocyanine (**ZnPc-I**) is a possible photosensitizer candidate for PDT applications. The **ZnPc-I** showed an antiproliferative effect in vitro on human nonsmall cell lung carcinoma cell lines A549 and H1299 in a dose-dependent manner. Even though it is necessary to investigate the molecular mechanisms of this antiproliferative effect of the **ZnPc-I** compound, the results herein indicate that the **ZnPc-I** compound has strong potential for nonsmall cell lung carcinoma treatment.

## Supplementary Information

Additional ^1^H NMR, ^13^C NMR, FTIR, and mass spectra for the new compounds (Figures S1–S24) are given in the supplementary information.

## 1. Materials

4-Mercaptobenzoic acid, 5-chloro-1-pentyn and 1,8-diazabicyclo [5.4.0]-undec-7-ene (DBU) was purchased from Sigma-Aldrich Chemical Co. The other chemicals were from commercial suppliers. The solvents were purified according to the known methods [1]. Some reagents, such as 2-azido-ethylacetate [2], 4-mercaptobenzoic acid methyl ester [3], and 3,6-dimercapto phthalonitrile [4], were prepared as in the reference cited.

## 2. Equipment

The ^1^H and ^13^C NMR spectra were employed on Agilent-vnmrs 400 (Agilent Technologies, Santa Clara, CA, USA) or Varian Mercury Plus 300 MHz (Varian Medical Systems, Palo Alto, CA, USA) spectrometers. FTIR and UV-vis spectra were obtained on a Perkin-Elmer UATR Two Spectrometer (PerkinElmer Inc., Waltham, MA, USA) and a Shimadzu 1800 spectrophotometer (Shimadzu Corp., Kyoto, Japan) using 1-cm path length cuvettes at room temperature, respectively. Fluorescence excitation and emission spectra were recorded on a Varian Eclipse spectrofluorometer using 1-cm path length cuvettes at room temperature. PerkinElmer Analyst 700 Atomic Absorption Spectrometer using the emission method (signal type) at λ = 285.2 nm for determination of the zinc. Elemental analyses and mass spectra were recorded on a Costech ECS 4010 analyzer (Costech Instruments, Milan, Italy) and Micromass Quatro liquid chromatography (LC)/Ultima LC/ tandem mass spectrometry (MS/MS) (Waters Corp., Milford, MA, USA) or Bruker Daltonicis Microflex LT MALDI-TOF spectrometer (Bruker Corp., Billerica, MA, USA). Melting points were measured on an Electrothermal 9100 apparatus (Bibby Scientific Ltd., Stone, Staffordshire, UK) in a sealed tube.

## 3. Photochemical parameter

### 3.1. Singlet oxygen quantum yield

Singlet oxygen quantum yield (Φ_Δ_) determination was performed according to the literature. Typically, a 3-mL portion of the respective studied phthalocyanine compound (**ZnPc-I**) solution (C = 1 × 10^−5^ M) containing the singlet oxygen quencher was irradiated in the Q band region with the photoirradiation set-up literature [5,6]. The Φ_Δ_ values were determined in the air using the relative method with unsubstituted ZnPc in DMSO as a standard. 1,3-Diphenylisobenzofuran (DPBF) was used as a chemical quencher for singlet oxygen produced during irradiation. Eq. (1) was employed for the determination of Φ_Δ_ value:


$$\Phi_{\Delta }=\Phi_{\Delta }^{\text{Std}}\frac{\text{R}.{\text{I}}_{\text{abs}}^{\text{Std}}}{{\text{R}}^{\text{Std}}.{\text{I}}_{\text{abs}}}$$

Here, $\Phi_{\Delta }^{\text{Std}}$ is the singlet oxygen quantum yield for the standard **ZnPc_std_** (Φ_Δ_=0.67 in DMSO) [7], R and R_Std_ are the DPBF photobleaching rates in the presence of the respective phthalocyanine compound (**ZnPc-I**) in DMSO and standard, respectively. I_abs_ and ${\text{I}}_{\text{abs}}^{\text{Std}}$ are the rates of light absorption by sample (**ZnPc-I**) and the standard, respectively. To avoid chain reactions induced by quenchers (DPBF) in the presence of singlet oxygen, the concentration of quencher (DPBF) was lowered to ~3 × 10^−5^ M [8]. The solution of a sensitizer (C = 1 × 10^−5^ M) containing a quencher (DPBF) was prepared in the dark and irradiated by the light around the Q-band of the studied phthalocyanines. The DPBF photooxidation was monitored at 417 nm. The light intensity 6.21 × 10^15^ photons s^−1^ cm^−2^ was used for Φ_Δ_ determination.

## 4. In vitro studies

### 4.1. Cell culture conditions

Human nonsmall cell lung carcinoma A549 cells were cultured in DMEM (Gibco Scientific, Grand Island, NY, USA) and H1299 cells were cultured in RPMI-1640 (Gibco Scientific) medium supplemented with 10% fetal bovine serum (Gibco Scientific) and 1% penicillin-streptomycin (Gibco Scientific), respectively, at 37 °C in a humidified atmosphere of 5% CO_2_.

### 4.2. Determination of the antiproliferative effect of the ZnPc-I compound

The cytotoxic activity of the **ZnPc-I** compound was determined by the following method of Akca et al. [9]. Briefly, the A549 and H1299 cells were seeded into a 96-well plate (3 ×10^3^ cells/well), and the cells were treated with different concentrations of the **ZnPc-I** compound (1000, 500, 250, 100, 50, 10, and 1 μg/mL). After 72 h of incubation, cytotoxicity was determined by the colorimetric method using MTT.

References1

LeznofCC
LeverABP

Phthalocyanines: Properties and Applications
New York, NY, USA: VCH,
1989
2

ChoiJ
LeeW
NamgoongJW
KimTM
KimJP

Synthesis and characterization of novel triazatetrabenzcorrole dyes for LCD color filter and black matrix
Dyes and Pigments
2013
99
357
365
10.1016/j.dyepig.2013.05.026
3

SoganciT
BayguY
KabayN
GökY
AkM

Comparative investigation of peripheral and nonperipheral zinc phthalocyanine-based polycarbazoles in terms of optical, electrical, and sensing properties
ACS Applied Materials & Interfaces
2018
10
21654
21665
10.1021/acsami.8b06206
29870222
4

MajeedSA
GhazalB
NevonenDE
GoffPC
BlankDA


Evaluation of the intramolecular charge-transfer properties in solvatochromic and electrochromic zinc octa(carbazolyl)phthalocyanines
Inorganic Chemistry
2017
56
11640
11653
10.1021/acs.inorgchem.7b01570
28920685
5

YiJ
ChenZ
XiangJ
ZhangF

Photocontrollable J-aggregation of a diarylethene-phthalocyanine hybrid and its aggregation-stabilized photochromic behavior
Langmuir
2011
27
8061
8066
10.1021/la201197k
21667954
6

BouvetM

Phthalocyanine-based field-effect transistors as gas sensors
Analytical and Bioanalytical Chemistry
2006
384
366
373
10.1007/s00216-005-3257-6
15933850
7

TomecekD
HruskaM
FitlP
VlcekJ
MaresovaE


Phthalocyanine photoregeneration for low power consumption chemiresistors
ACS Sensors
2018
3
2558
2565
10.1021/acssensors.8b00922
30431256
8

BayguY
CapanR
ErdoganM
OzkayaC
AcikbasY


Synthesis, characterization and chemical sensor properties of a novel Zn(II) phthalocyanine containing 15-membered dioxa-dithia macrocycle moiety
Synthetic Metals
2021
280
10.1016/j.synthmet.2021.116870
9

YangF
ForrestSR

Photocurrent generation in nanostructured organic solar cells
ACS Nano
2008
2
1022
1032
10.1021/nn700447t
19206500
10

HeJ
BenköG
KorodiF
PolívkaT
LomothR


Modified phthalocyanines for efficient near-IR sensitization of nanostructured TiO 2 electrode
Journal of the American Chemical Society
2002
124
4922
4932
10.1021/ja0178012
11971744
11

InceM
YumJH
KimY
MathewS
GrätzelM


Molecular engineering of phthalocyanine sensitizers for dye-sensitized solar cells
The Journal of Physical Chemistry C
2014
118
17166
17170
10.1021/jp502447y
12

YıldızB
GüzelE
MengesN
Şişmanİ
Kasım ŞenerM

Pyrazole-3-carboxylic acid as a new anchoring group for phthalocyanine-sensitized solar cells
Solar Energy
2018
174
527
536
10.1016/j.solener.2018.09.039
13

ZhangFL
SongMR
YuanGK
YeHN
TianY


A molecular combination of zinc(II) phthalocyanine and tamoxifen derivative for dual targeting photodynamic therapy and hormone therapy
Journal of Medicinal Chemistry
2017
60
6693
6703
10.1021/acs.jmedchem.7b00682
28699738
14

GüzelE

Dual-purpose zinc and silicon complexes of 1,2,3-triazole group substituted phthalocyanine photosensitizers: Synthesis and evaluation of photophysical, singlet oxygen generation, electrochemical and photovoltaic properties
RSC Advances
2019
9
10854
10864
10.1039/c8ra10665g
35515285
PMC906264215

TuncelS
DumoulinF
GailerJ
SooriyaarachchiM
AtillaD


A set of highly water-soluble tetraethyleneglycol-substituted Zn(ii) phthalocyanines: synthesis, photochemical and photophysical properties, interaction with plasma proteins and in vitro phototoxicity
Journal of the Chemical Society, Dalton Transactions
2011
40
4067
4079
10.1039/c0dt01260b
2115265516

OgunsipeA
ChenJY
NyokongT

Photophysical and photochemical studies of zinc(II) phthalocyanine derivatives - Effects of substituents and solvents
New Journal of Chemistry
2004
28
822
827
10.1039/b315319c
17

ChenJ
ChenN
HuangJ
WangJ
HuangM

Derivatizable phthalocyanine with single carboxyl group: synthesis and purification
Inorganic Chemistry Communications
2006
9
313
315
10.1016/j.inoche.2005.12.002
18

AhsenV
YilmazerE
ErtasM
BekâroğluÖ

Synthesis and characterization of metal-free and metal derivatives of a novel soluble crown-ether-containing phthalocyanine
Journal of the Chemical Society, Dalton Transactions
1988
401
406
10.1039/DT9880000401
19

DilberG
AltunparmakH
NasA
KantekinH
DurmuşM

The peripheral and non-peripheral 2H-benzotriazole substituted phthalocyanines: Synthesis, characterization, photophysical and photochemical studies of zinc derivatives
Spectrochimica Acta Part A: Molecular and Biomolecular Spectroscopy
2019
217
128
140
10.1016/j.saa.2019.01.095
30928838
20

HeH
LiuJY
NgDKP

A phthalocyanine-based fluorescent sensor for Zn2+ ion
Journal of Porphyrins and Phthalocyanines
2013
17
99
103
10.1142/S1088424612501374
21

BächleF
SiemensN
ZieglerT

Glycoconjugated Phthalocyanines as Photosensitizers for PDT – Overcoming Aggregation in Solution
European Journal Organic Chemistry
2019
2019
7089
7116
10.1002/ejoc.201901224
22

KantekinH
SarkıG
Ömeroğluİ
YalazanH
KahrimanN


Synthesis of peripheral and non-peripheral substituted metallophthalocyanines containing (E)-3-(5-bromo-2-hydroxphenyl)-1-o-tolyprop-2-en-1-one: Investigation of the photophysical and photochemical properties
Spectrochimica Acta Part A: Molecular and Biomolecular Spectroscopy
2021
252
119474
10.1016/j.saa.2021.119474
33517218
23

JosefsenLB
BoyleRW

Photodynamic therapy and the development of metal-based photosensitisers
Metal-Based Drugs 2008
2008
10.1155/2008/276109
PMC25358271881561724

XuWT
HuangB
DaiJJ
XuJ
XuHJ

Catalyst-free singlet oxygen-promoted decarboxylative amidation of α-keto acids with free amines
Organic Letters
2016
18
3114
3117
10.1021/acs.orglett.6b01296
27305073
25

GünselA
BilgiçliAT
TüzünB
PişkinH
AtmacaGY


Synthesis of tetra-substituted phthalocyanines bearing 2-(ethyl(m-tolyl)amino)ethanol: Computational and photophysicochemical studies
Journal of Photochemistry and Photobiology A: Chemistry
2019
373
77
86
10.1016/j.jphotochem.2018.12.038
26

ÇelikÇ
FarajzadehN
AkınM
AtmacaGY
SağlamÖ


Investigation of the biological and photophysicochemical properties of new non-peripheral fluorinated phthalocyanines
Dalton Transactions
2021
50
2736
2745
10.1039/d0dt04351f
33533372
27

TilloA
StolarskaM
KryjewskiM
PopendaL
SobottaL


Phthalocyanines with bulky substituents at non-peripheral positions - Synthesis and physico-chemical properties
Dyes and Pigments
2016
127
110
115
10.1016/j.dyepig.2015.12.017
28

KırbaçE
ErdoğmuşA

New non-peripherally substituted zinc phthalocyanines; synthesis, and comparative photophysicochemical properties
Journal of Molecular Structure
2020
1202
127392
10.1016/j.molstruc.2019.127392
29

MbambisaG
TauP
AntunesE
NyokongT

Synthesis and electrochemical properties of purple manganese(III) and red titanium(IV) phthalocyanine complexes octa-substituted at non-peripheral positions with pentylthio groups
Polyhedron
2007
26
5355
5364
10.1016/j.poly.2007.08.007
30

Aktas KamilogluA
OmerogluI
YalazanH
DurmusM
CelikG


Photophysical, photochemical properties of chalcone substituted Zinc(II) and Magnesium(II) metallophthalocyanines bearing thiophene units
Journal of Inclusion Phenomena and Macrocyclic Chemistry
2022
102
693
703
10.1007/s10847-022-01152-3
31

SvecJ
ZimcikP
NovakovaL
RakitinOA
AmelichevSA


1,2,5-chalcogenadiazole-annulated tripyrazinoporphyrazines: synthesis, spectral characteristics, and influence of the heavy atom effect on their photophysical properties
European Journal of Organic Chemistry
2015
2015
596
604
10.1002/ejoc.201403329
32

KobayashiN
FuruyamaT
SatohK

Rationally designed phthalocyanines having their main absorption band beyond 1000 nm
Journal of the American Chemical Society
2011
133
19642
19645
10.1021/ja208481q
22074492
33

FuruyamaT
SatohK
KushiyaT
KobayashiN

Design, synthesis, and properties of phthalocyanine complexes with main-group elements showing main absorption and fluorescence beyond 1000 nm
Journal of the American Chemical Society
2014
136
765
776
10.1021/ja411016f
24328229
34

GürolI
DurmuşM
AhsenV
NyokongT

Synthesis, photophysical and photochemical properties of substituted zinc phthalocyanines
Dalton Transactions
2007
3782
3791
10.1039/b704345g
17712444
35

DemirbaşÜ
Ömeroğluİ
AkçayHT
DurmuşM
KantekinH

Synthesis, characterization, photophysical and photochemical properties of peripherally tetra benzodioxane substituted metal-free phthalocyanine and its zinc(II) and magnesium(II) derivatives
Journal of Molecular Structure
2021
1223
128992
10.1016/j.molstruc.2020.128992
36

BayguY
GökY

Synthesis and characterization of new partially-aggregated water-soluble polyether-triazole linked zinc(II) phthalocyanines as photosensitizers for PDT studies
Synthetic Metals
2020
260
116256
10.1016/j.synthmet.2019.116256
37

DurmuşM
YamanH
GölC
AhsenV
NyokongT

Water-soluble quaternized mercaptopyridine-substituted zinc-phthalocyanines: Synthesis, photophysical, photochemical and bovine serum albumin binding properties
Dyes and Pigments
2011
91
153
163
10.1016/j.dyepig.2011.02.007
38

YurttaşAG
SevimAM
ÇınarK
AtmacaGY
ErdoğmuşA


The effects of zinc(II)phthalocyanine photosensitizers on biological activities of epitheloid cervix carcinoma cells and precise determination of absorbed fluence at a specific wavelength
Dyes and Pigments
2022
198
10.1016/j.dyepig.2021.110012


## 5. FTIR, mass, NMR, and UV-vis spectrums of novel compounds

Figure S1 FTIR spectrum of compound **3**.

Figure S2 Mass spectrum of compound **3**.

Figure S3 ^1^H NMR spectrum of compound **3**.

Figure S4 ^13^C NMR spectrum of compound **3**.

Figure S5 FTIR spectrum of compound **4**.

Figure S6 Mass spectrum of compound **4**.

Figure S7 ^1^H NMR spectrum of compound **4**.

Figure S8 ^13^C NMR spectrum of compound **4**.

Figure S9 FTIR spectrum of compound **6**.

Figure S10 ^1^H NMR spectrum of compound **6**.

Figure S11 ^13^C NMR spectrum of compound **6**.

Figure S12 FTIR spectrum of compound **7**.

Figure S13 Mass spectrum of compound **7**.

Figure S14 ^1^H NMR spectrum of compound **7**.

Figure S15 ^13^C NMR spectrum of compound **7**.

Figure S16 FTIR spectrum of compound **9**.

Figure S17 ^1^H NMR spectrum of compound **9**.

Figure S18 ^13^C NMR spectrum of compound **9**.

Figure S19 Mass spectrum of compound **9**.

Figure S20 ^1^H NMR spectrum of compound **ZnPc-I**.

Figure S21 ^13^C NMR spectrum of compound **ZnPc-I**.

Figure S22 FTIR spectrum of compound **ZnPc-I**.

Figure S23 Mass spectrum of compound **ZnPc-I**.

Figure S24 UV-vis spectrum of compound **ZnPc-I** (1×10^−5^ M in DMSO).

## Figures and Tables

**Scheme:**
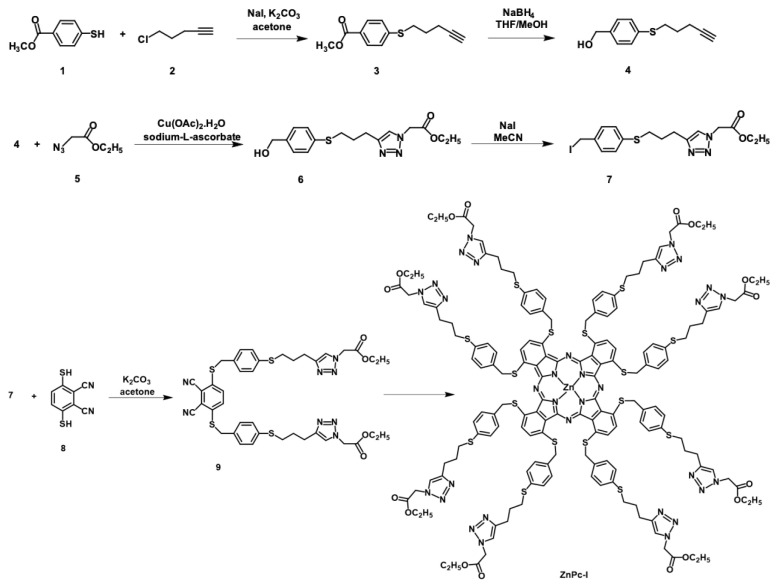
Synthetic procedures of **ZnPc-I**.

**Table:** Absorption, emission, and excitation spectral data and photochemical parameter of zinc(II) phthalocyanine (**ZnPc-I**) in DMSO.

Compound	Absorbance λ_max_ (nm)	(log ɛ)	Emission λ_Em_ (nm)	Excitation λ_Ex_ (nm)	Stokes shift Δ_Stokes_ (nm)	Φ_Δ_
**ZnPc-I**	789	4.75	-	-	-	0.66
**Unsub.ZnPc**	672^a^	5.14 a	672 a	682 a	10 a	0.67 b

a Data from Gürol et al. [34]

b data from Ogunsipe et al. [16].
