# Supplementary figures and images for: Synthesis, characterization, and investigation of photochemical and in vitro properties of novel Zn(II) phthalocyanine
Source: Turk J Chem. 2024 Oct 25;48(6):800–8. doi: 10.55730/1300-0527.3699 (PMC11706298; doi:10.55730/1300-0527.3699)

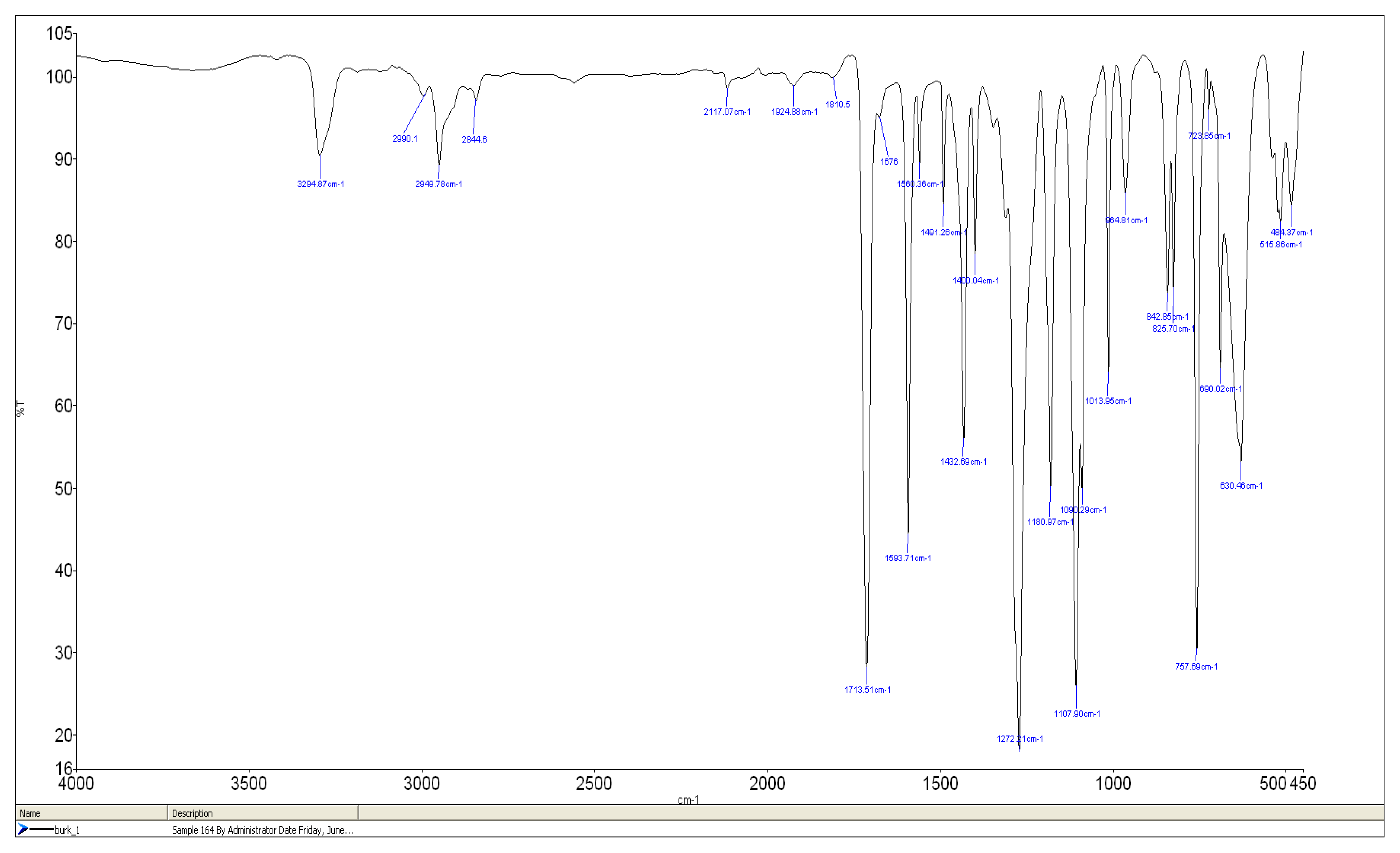

Supplement: Figure S1 — FTIR spectrum of compound 3. [file tjc-48-06-800s1.tif]

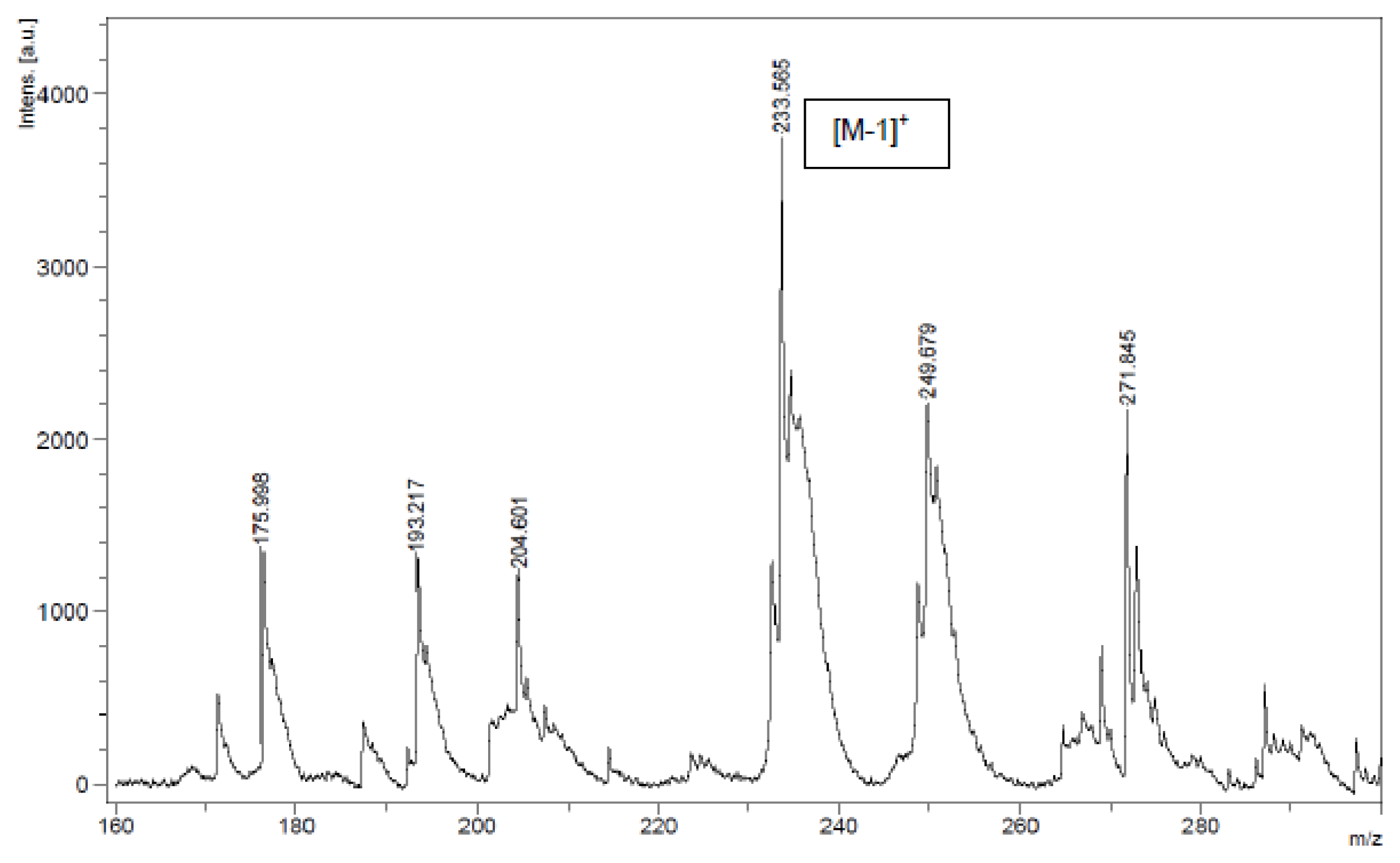

Supplement: Figure S2 — Mass spectrum of compound 3. [file tjc-48-06-800s2.tif]

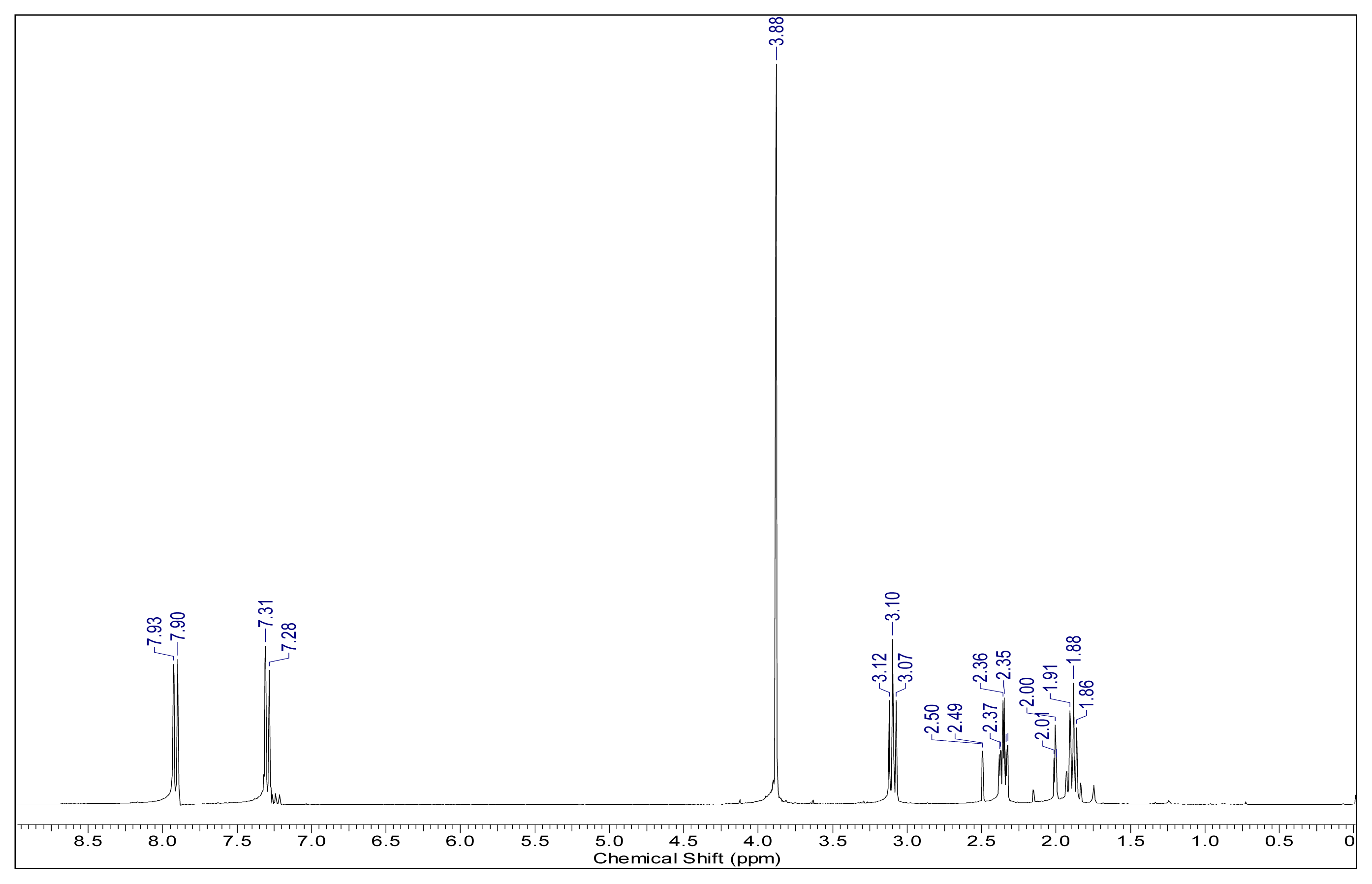

Supplement: Figure S3 — 1H NMR spectrum of compound 3. [file tjc-48-06-800s3.tif]

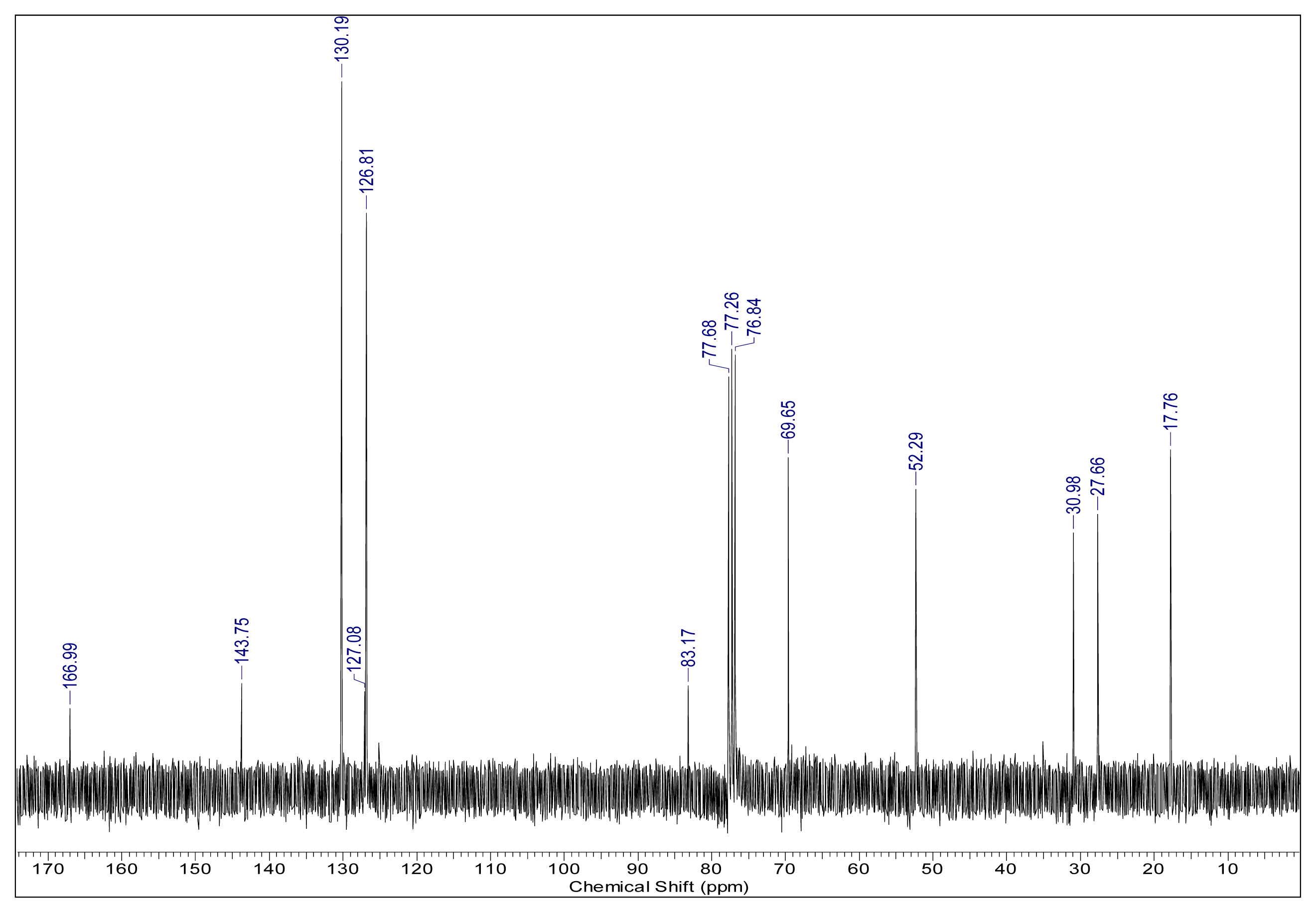

Supplement: Figure S4 — 13C NMR spectrum of compound 3. [file tjc-48-06-800s4.tif]

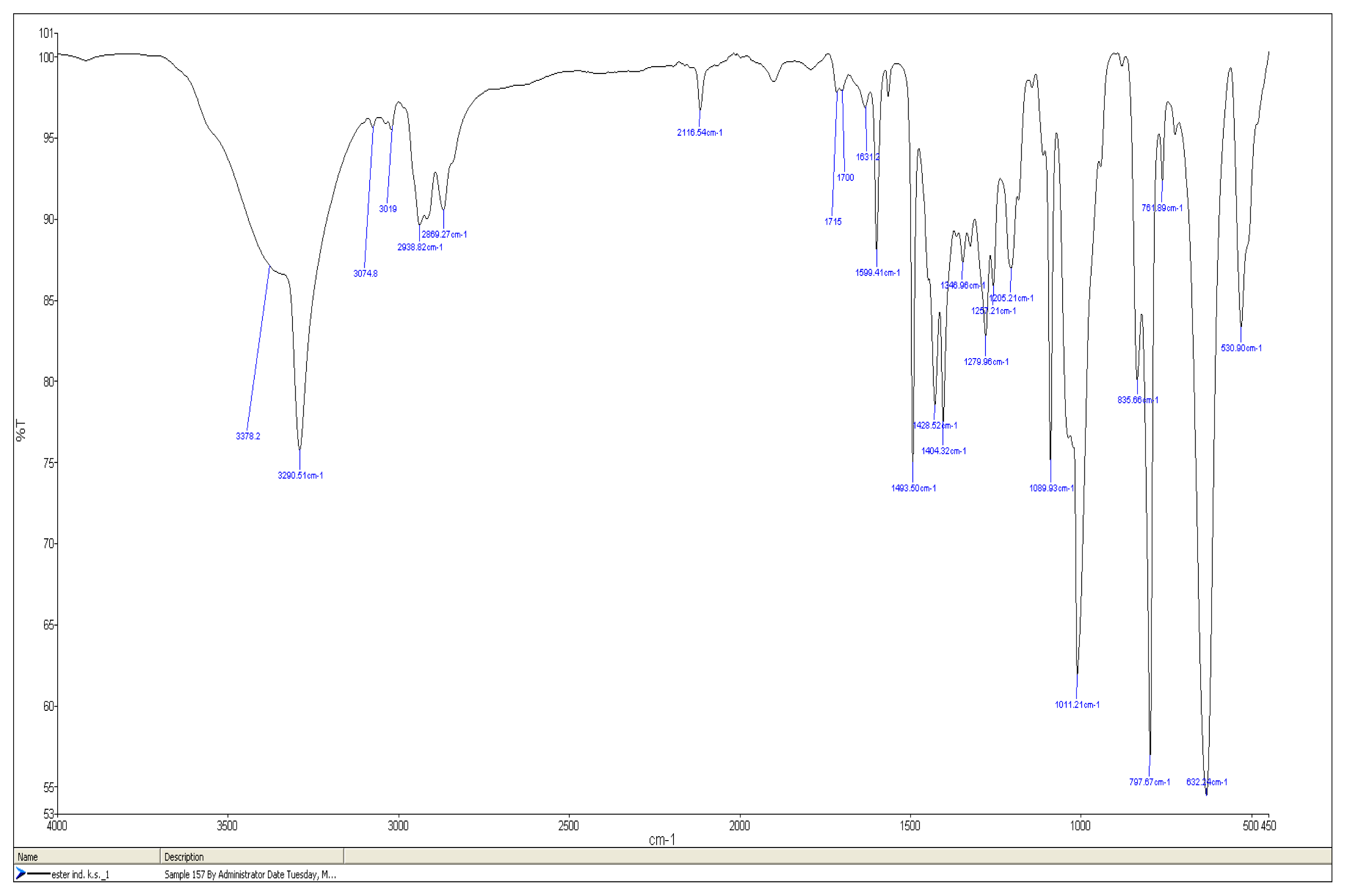

Supplement: Figure S5 — FTIR spectrum of compound 4. [file tjc-48-06-800s5.tif]

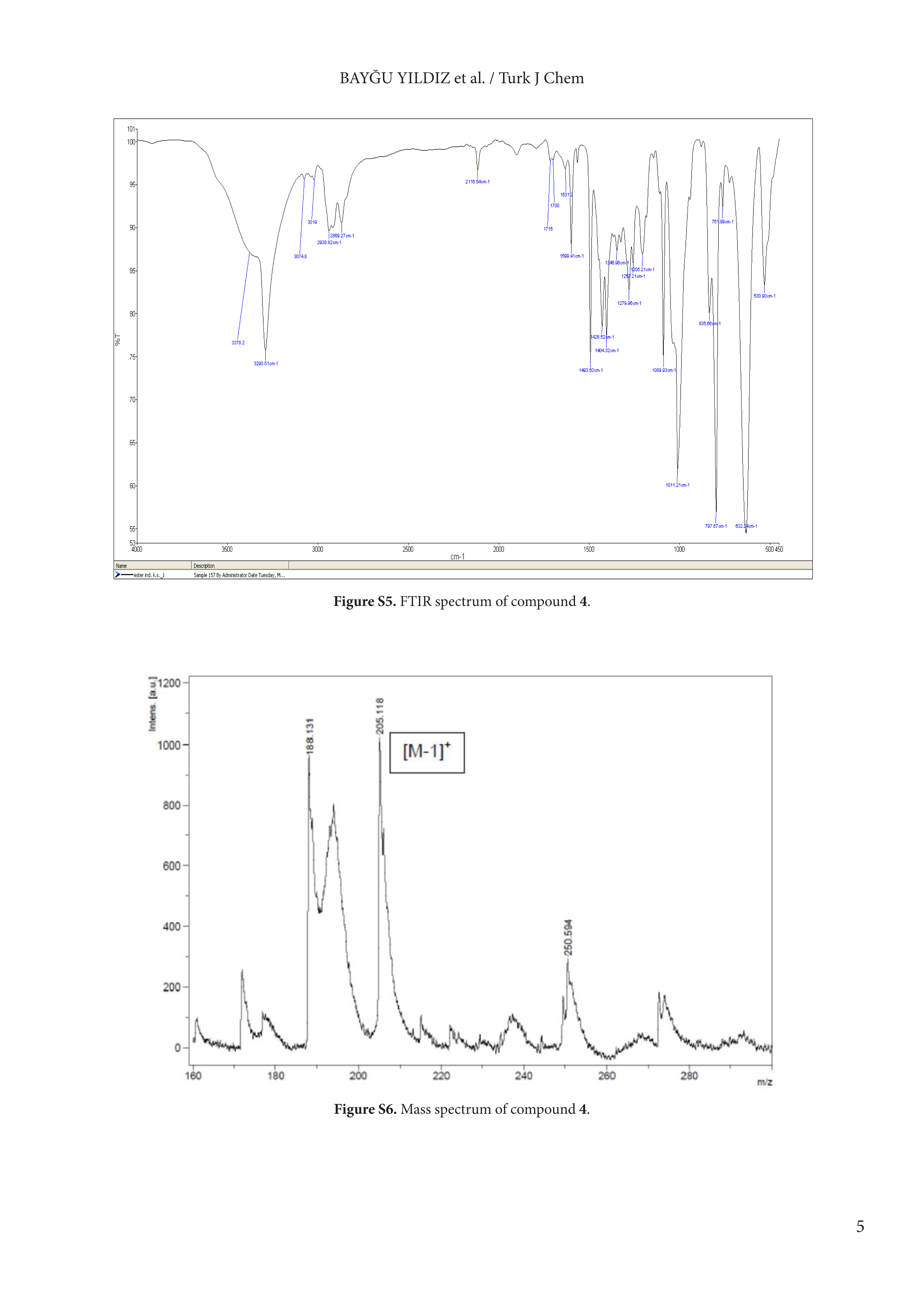

Supplement: Figure S6 — Mass spectrum of compound 4. [file tjc-48-06-800s6.tif]

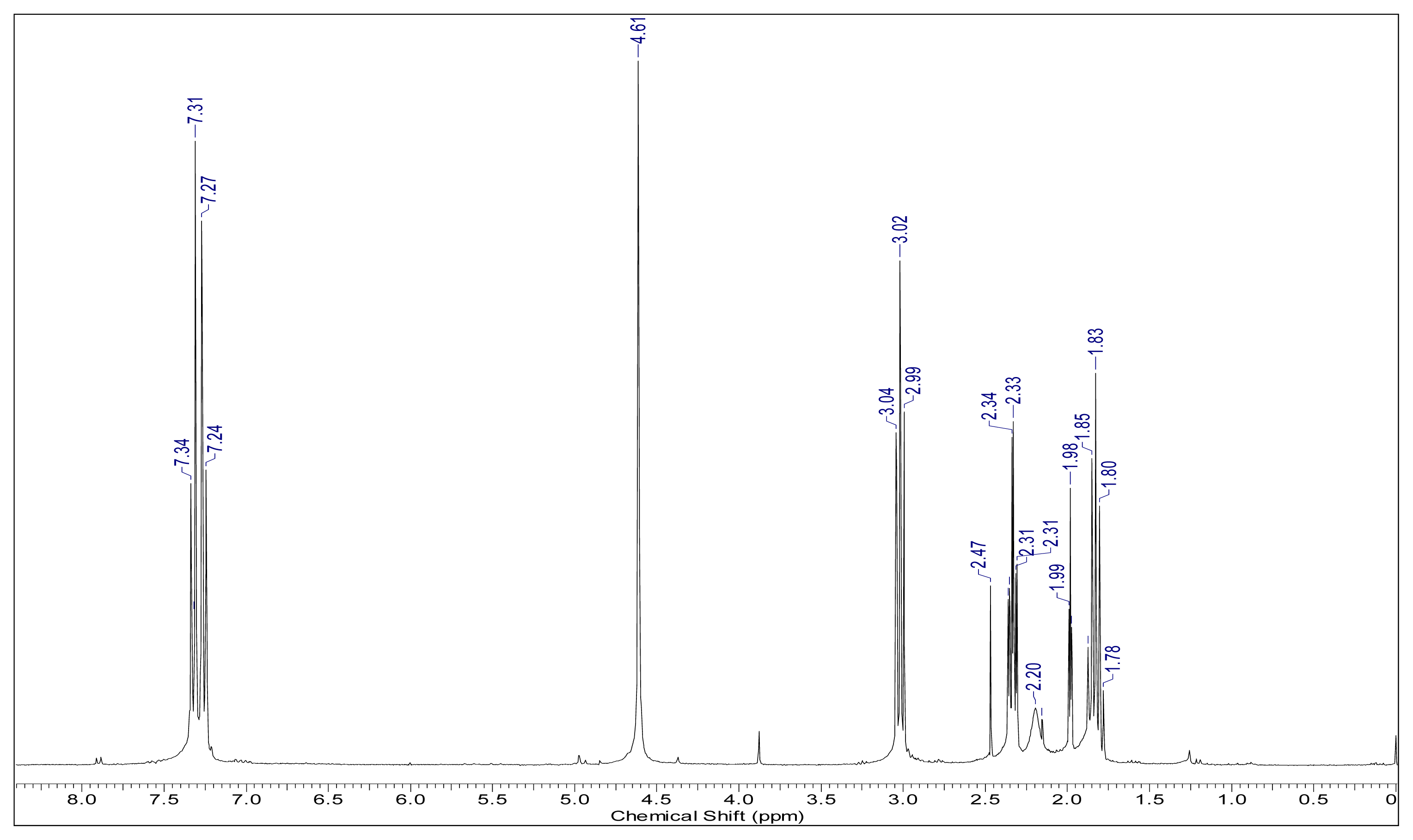

Supplement: Figure S7 — 1H NMR spectrum of compound 4. [file tjc-48-06-800s7.tif]

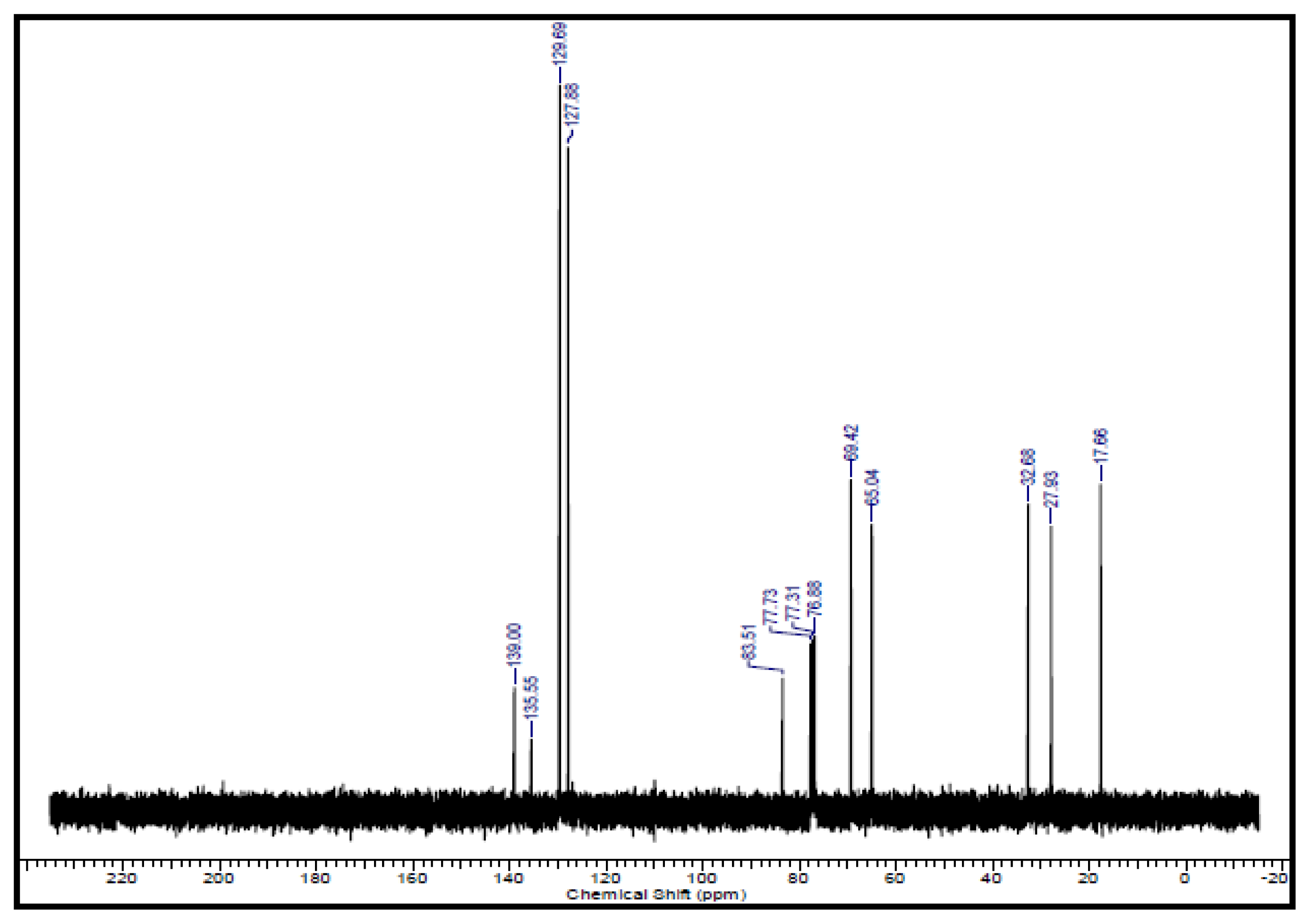

Supplement: Figure S8 — 13C NMR spectrum of compound 4. [file tjc-48-06-800s8.tif]

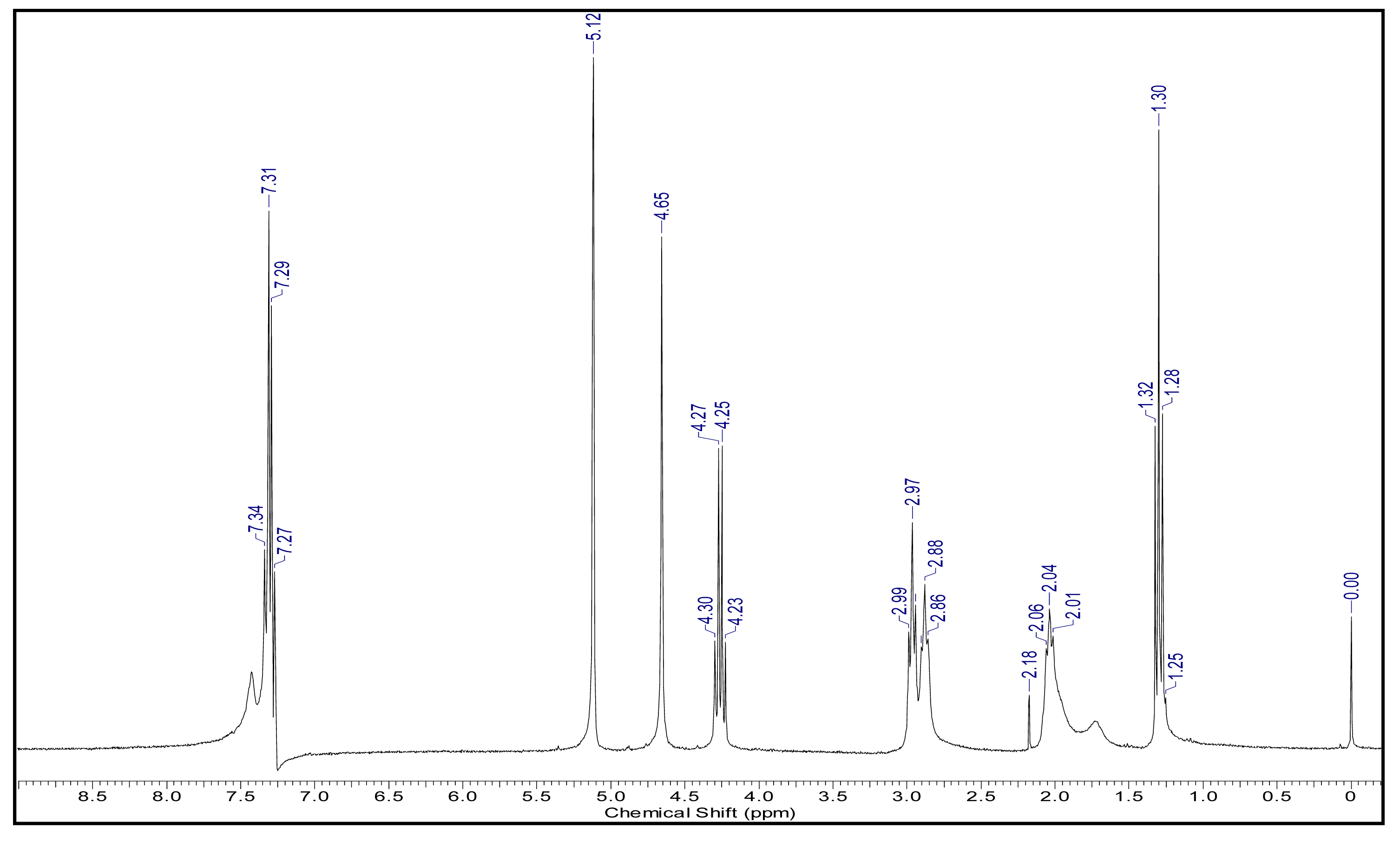

Supplement: Figure S9 — FTIR spectrum of compound 6. [file tjc-48-06-800s9.tif]

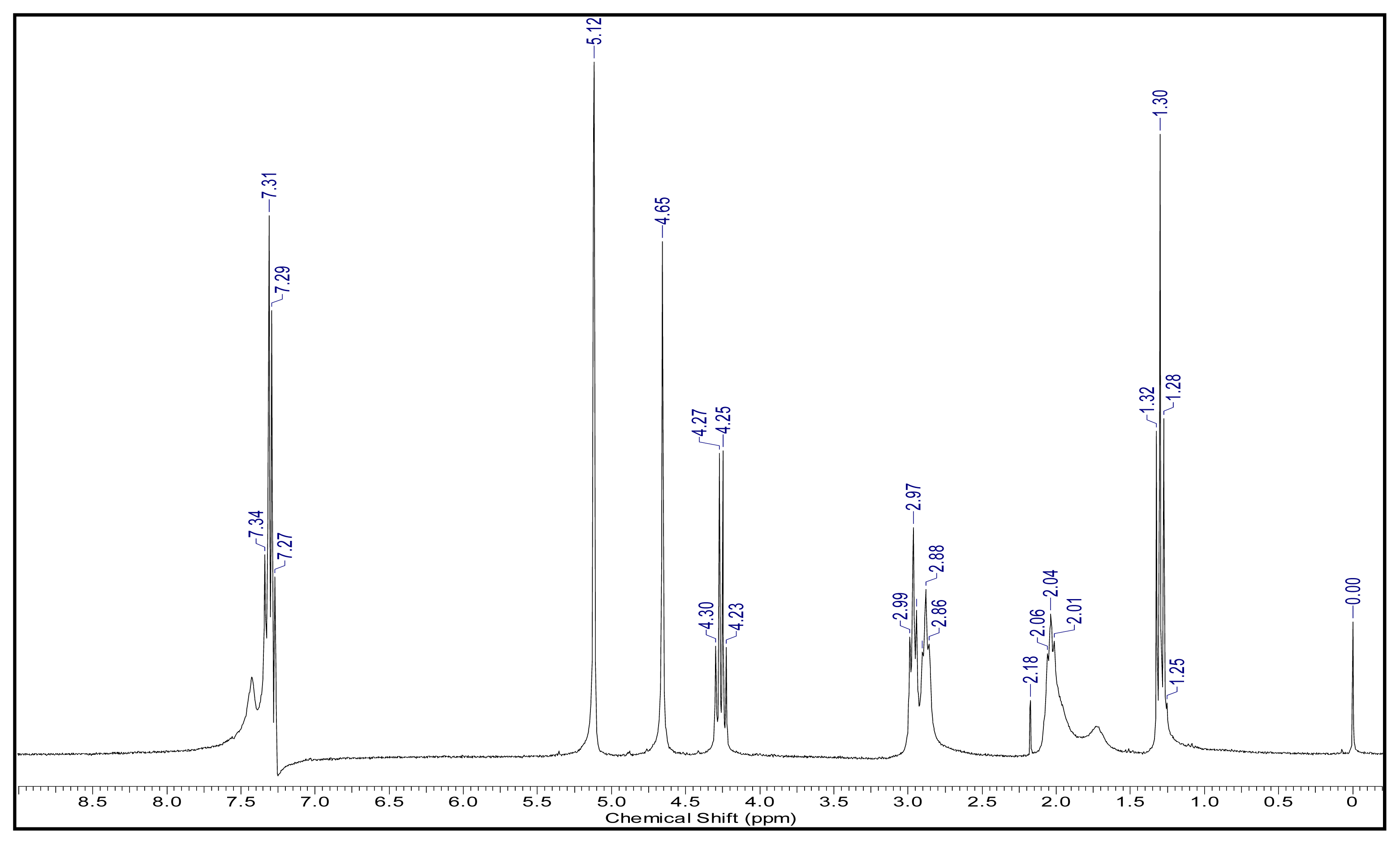

Supplement: Figure S10 — 1H NMR spectrum of compound 6. [file tjc-48-06-800s10.tif]

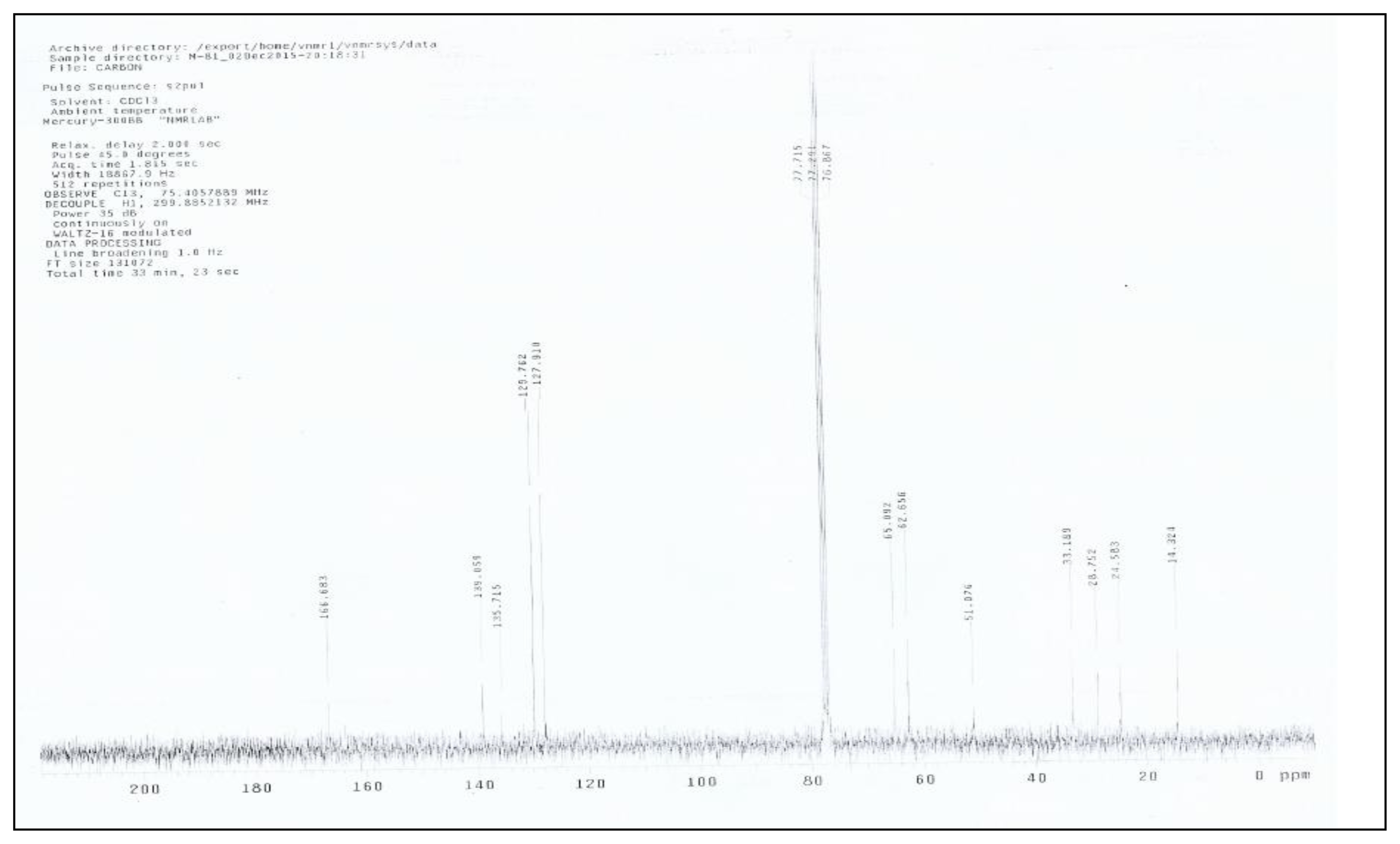

Supplement: Figure S11 — 13C NMR spectrum of compound 6. [file tjc-48-06-800s11.tif]

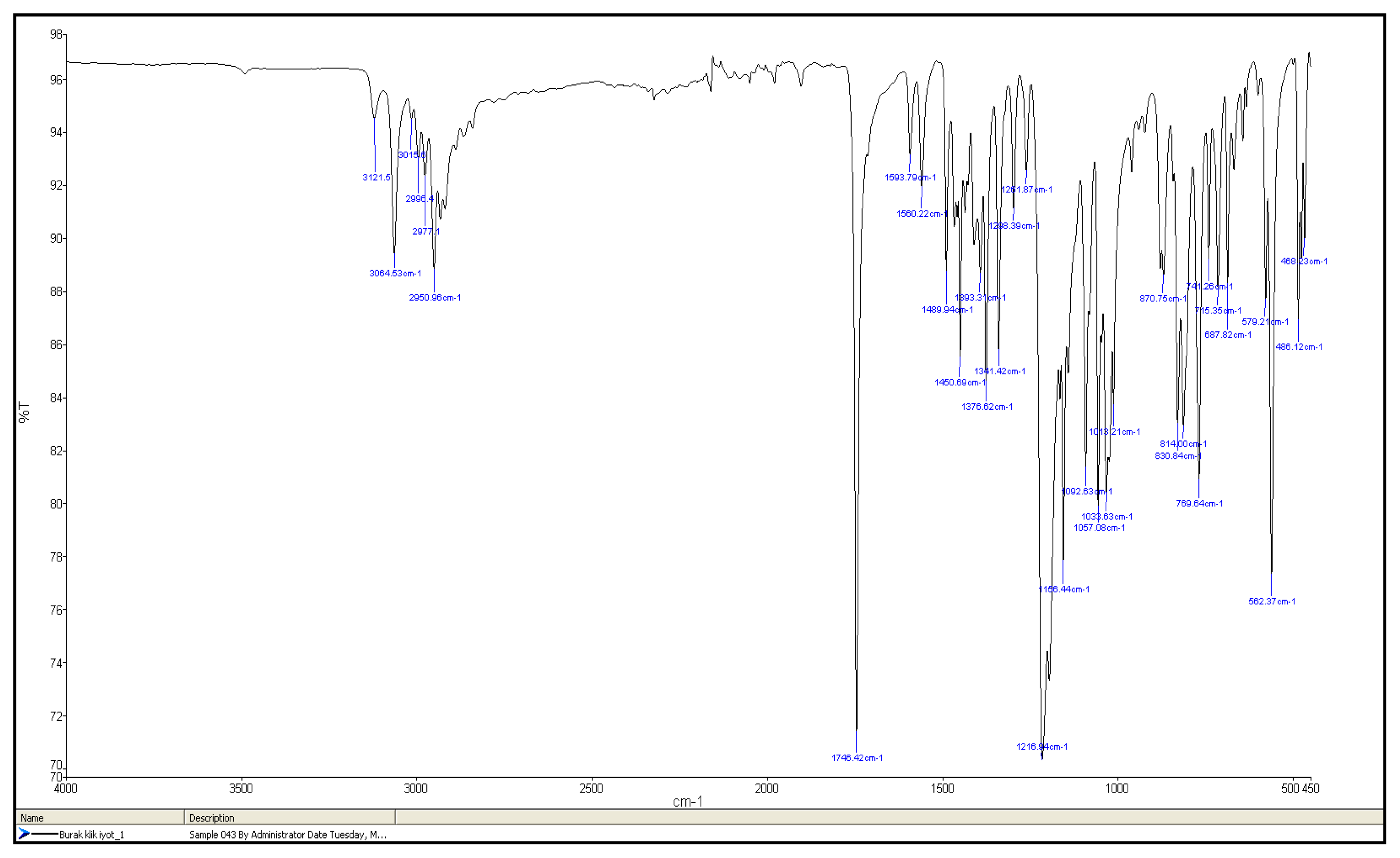

Supplement: Figure S12 — FTIR spectrum of compound 7. [file tjc-48-06-800s12.tif]

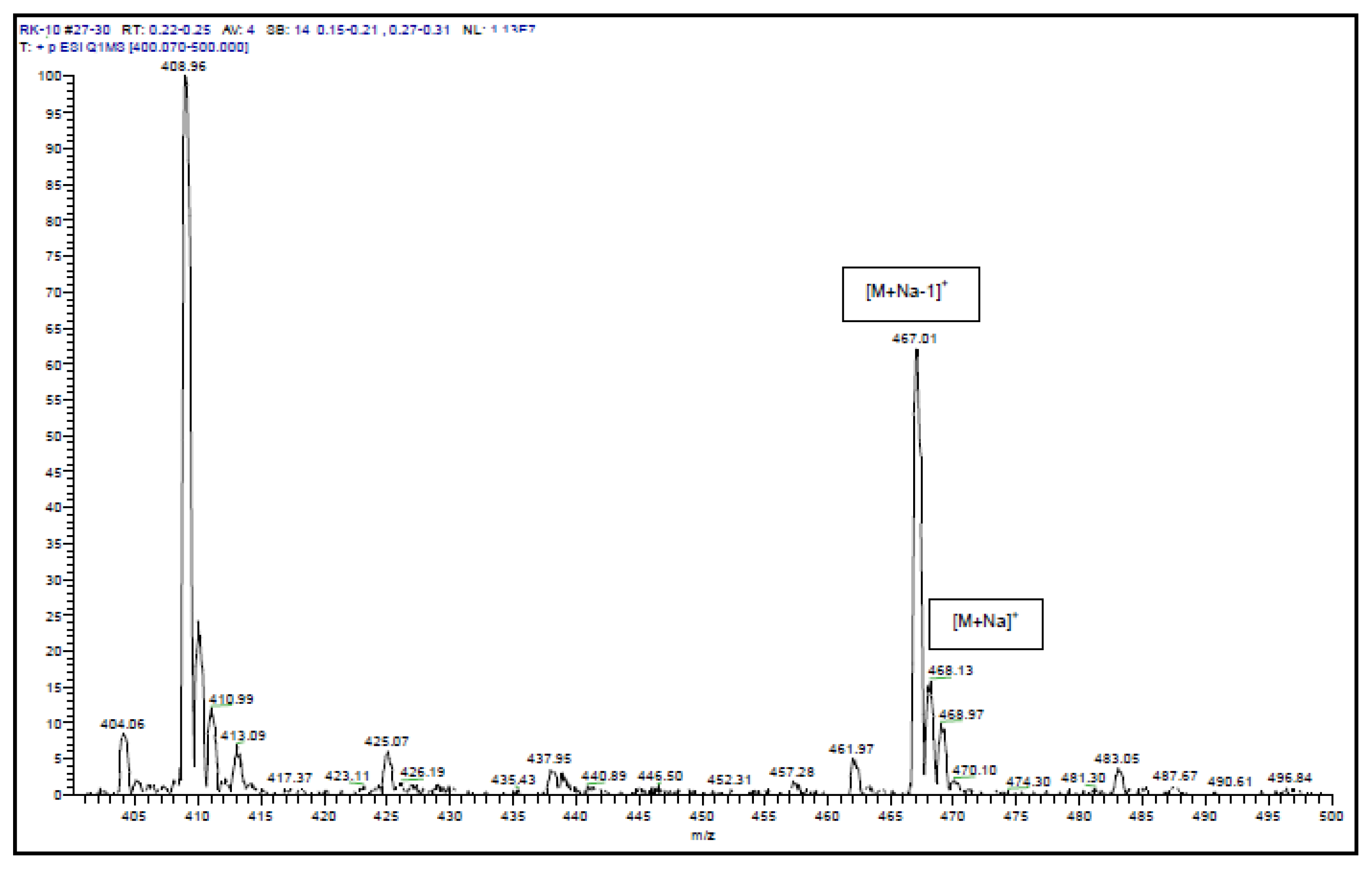

Supplement: Figure S13 — Mass spectrum of compound 7. [file tjc-48-06-800s13.tif]

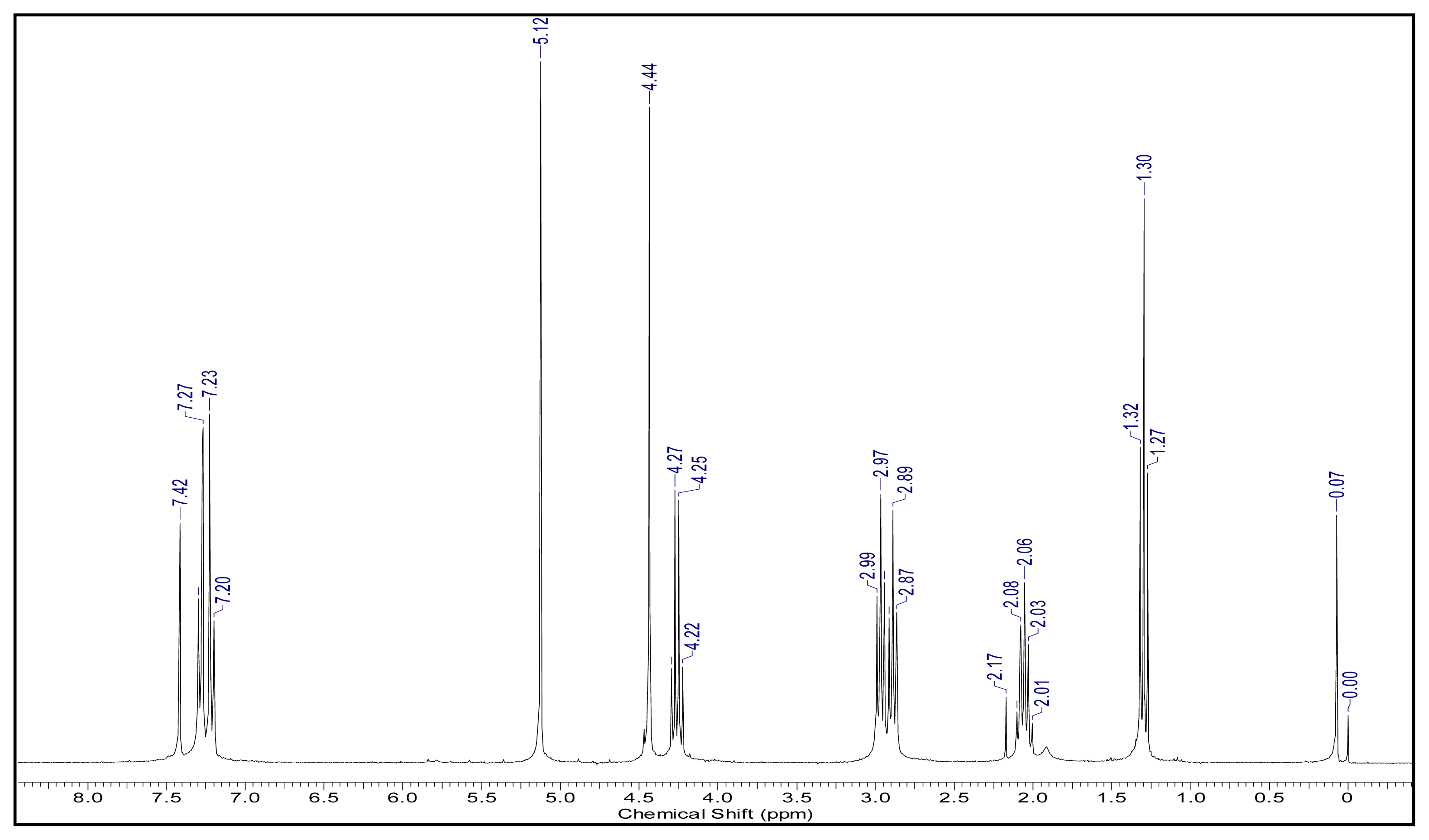

Supplement: Figure S14 — 1H NMR spectrum of compound 7. [file tjc-48-06-800s14.tif]

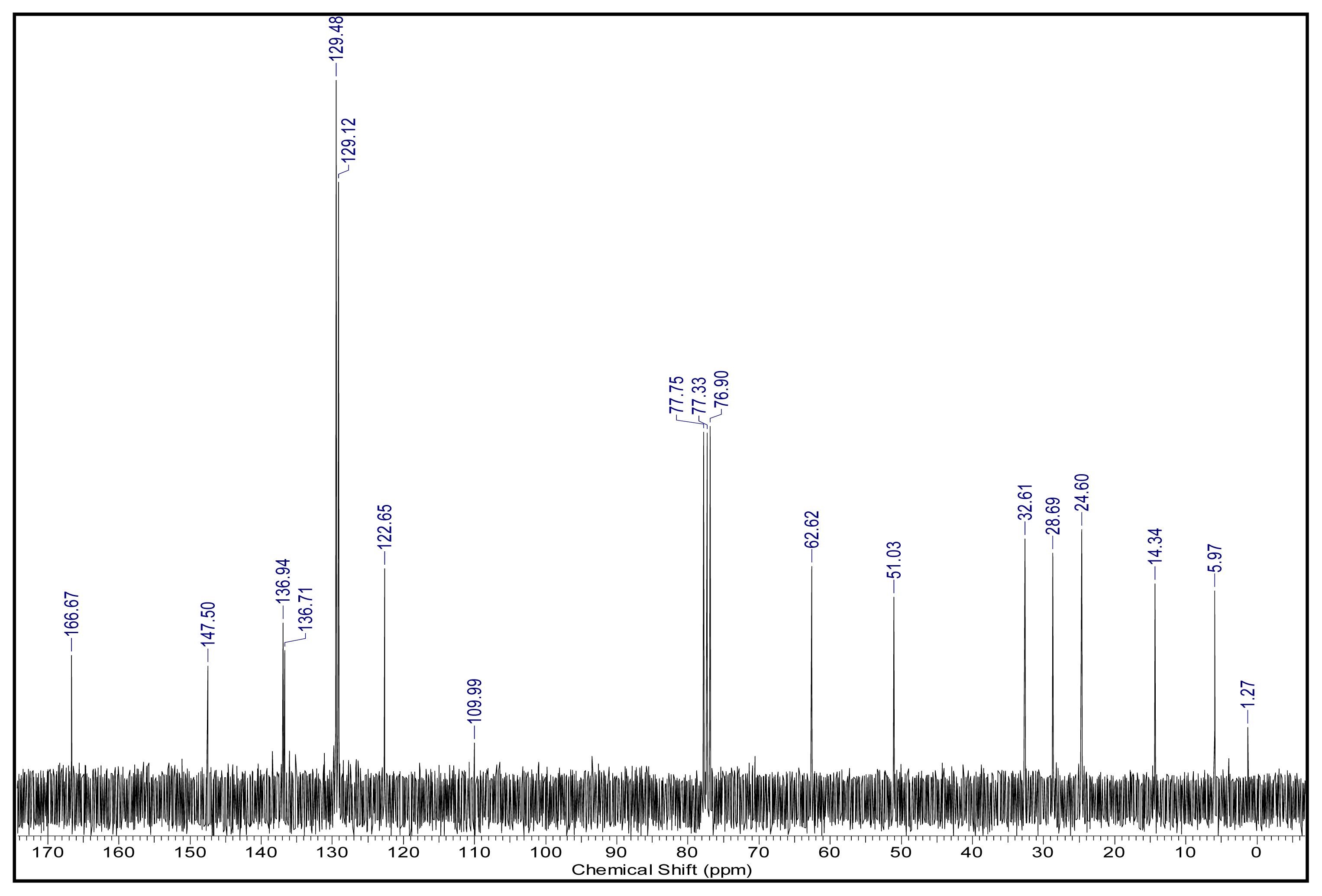

Supplement: Figure S15 — 13C NMR spectrum of compound 7. [file tjc-48-06-800s15.tif]

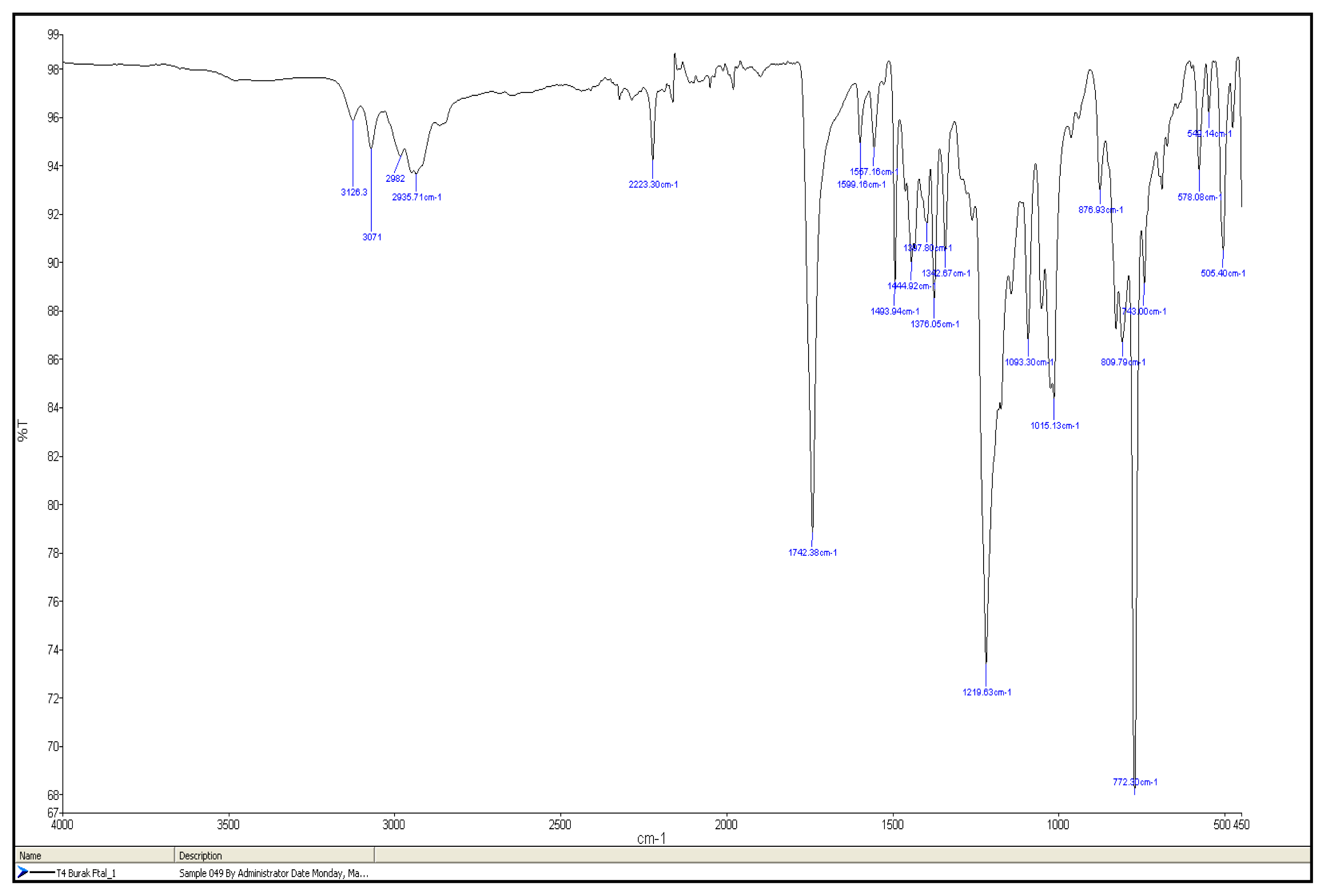

Supplement: Figure S16 — FTIR spectrum of compound 9. [file tjc-48-06-800s16.tif]

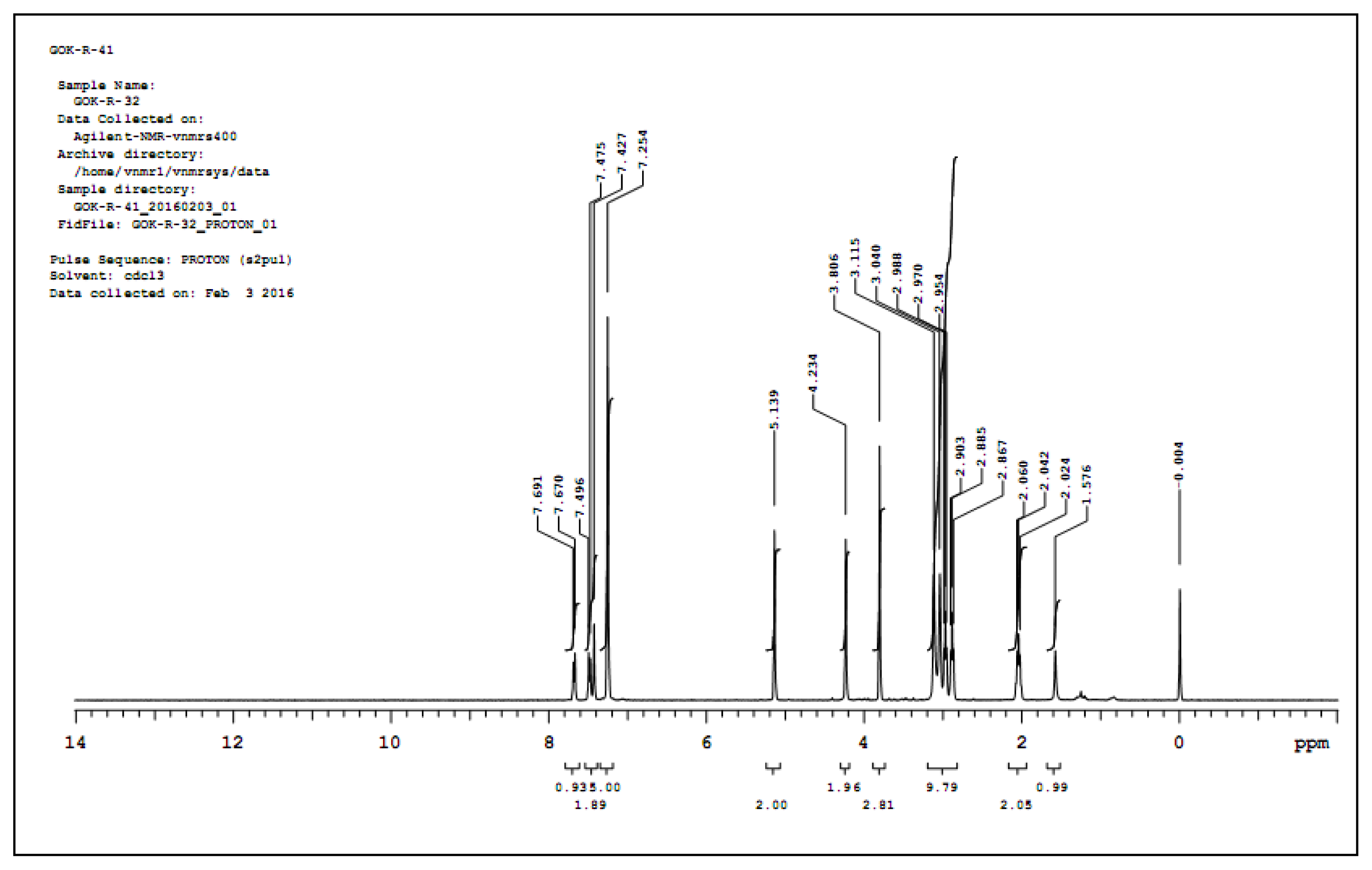

Supplement: Figure S17 — 1H NMR spectrum of compound 9. [file tjc-48-06-800s17.tif]

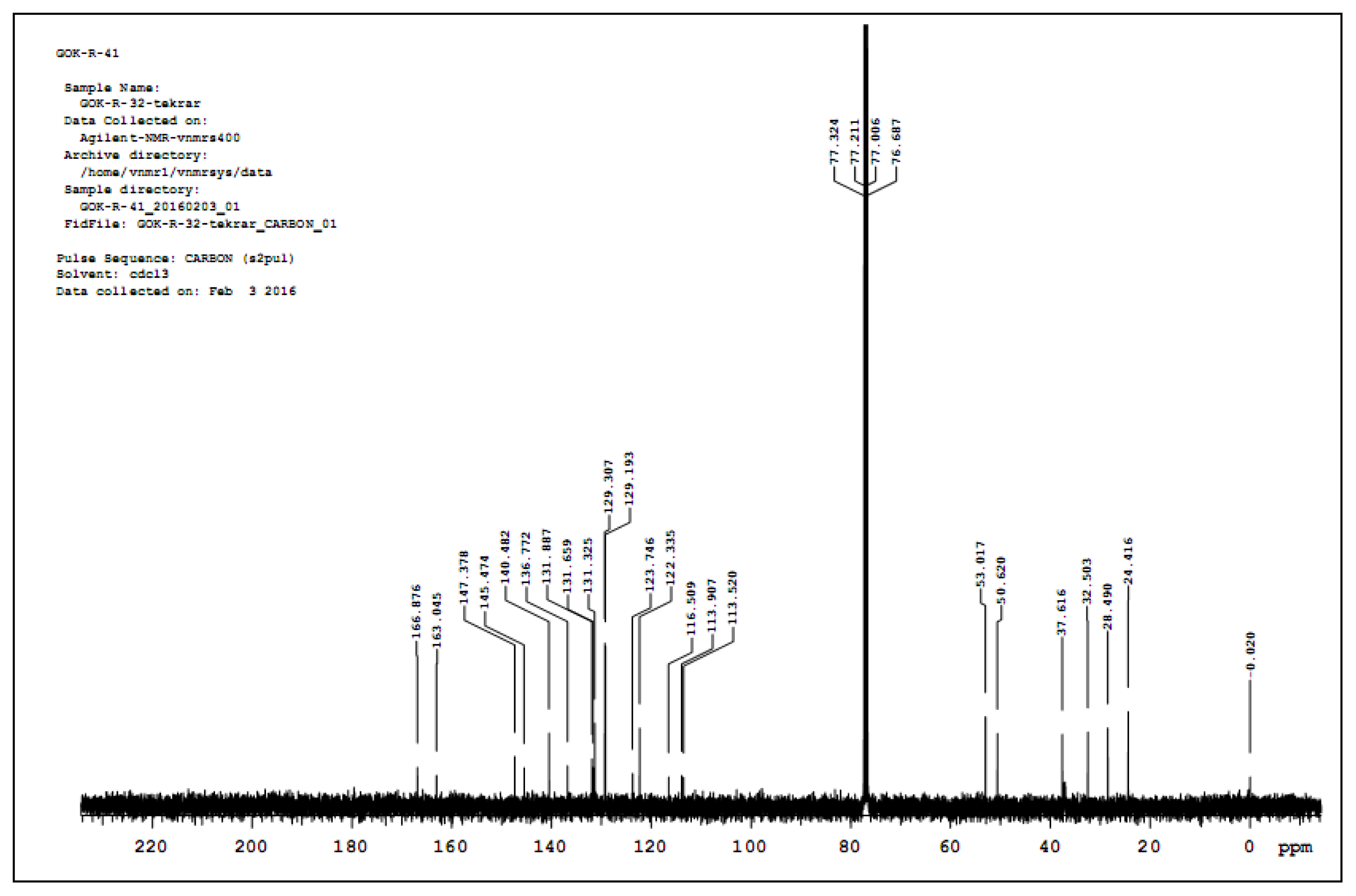

Supplement: Figure S18 — 13C NMR spectrum of compound 9. [file tjc-48-06-800s18.tif]

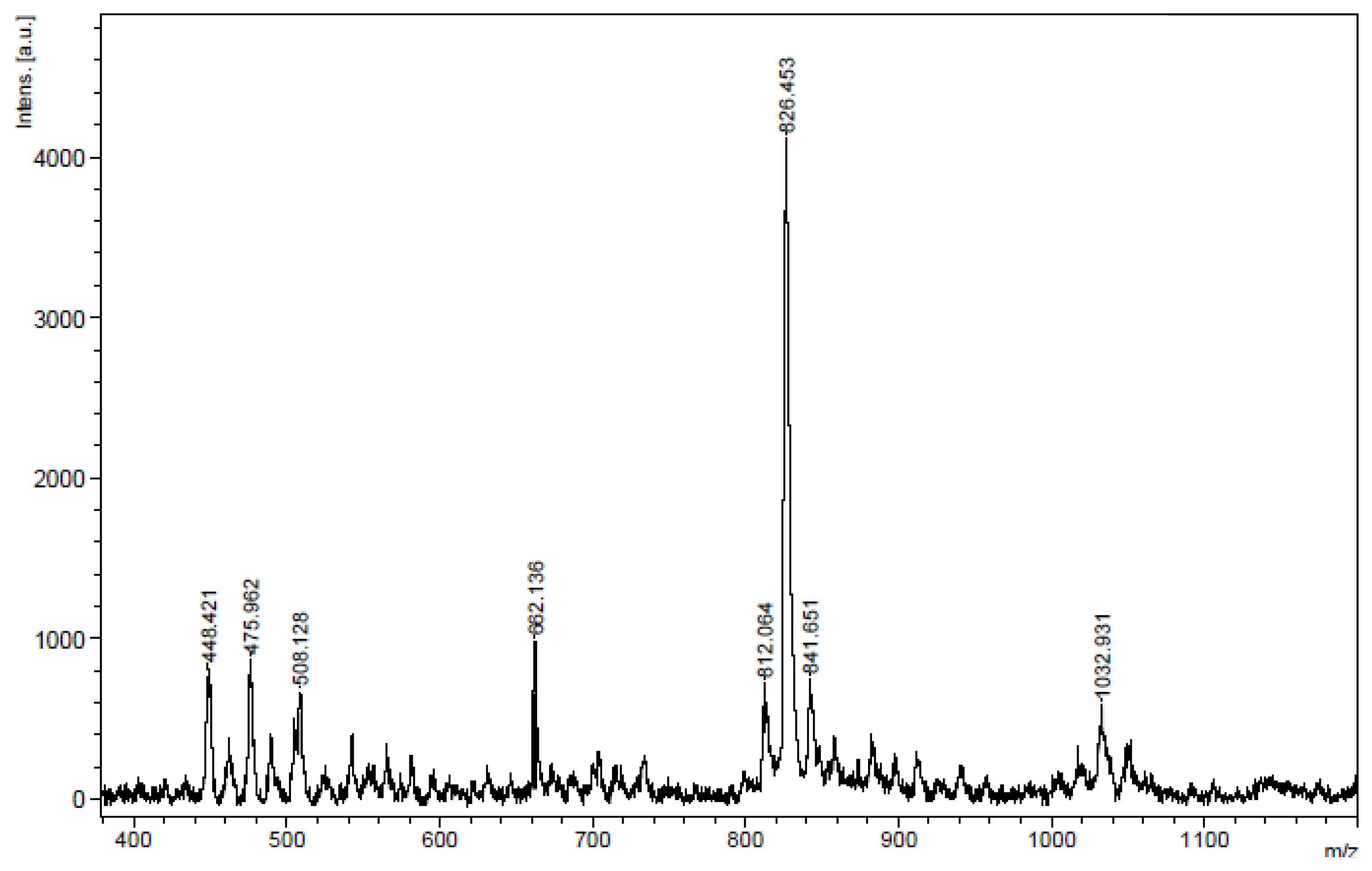

Supplement: Figure S19 — Mass spectrum of compound 9. [file tjc-48-06-800s19.tif]

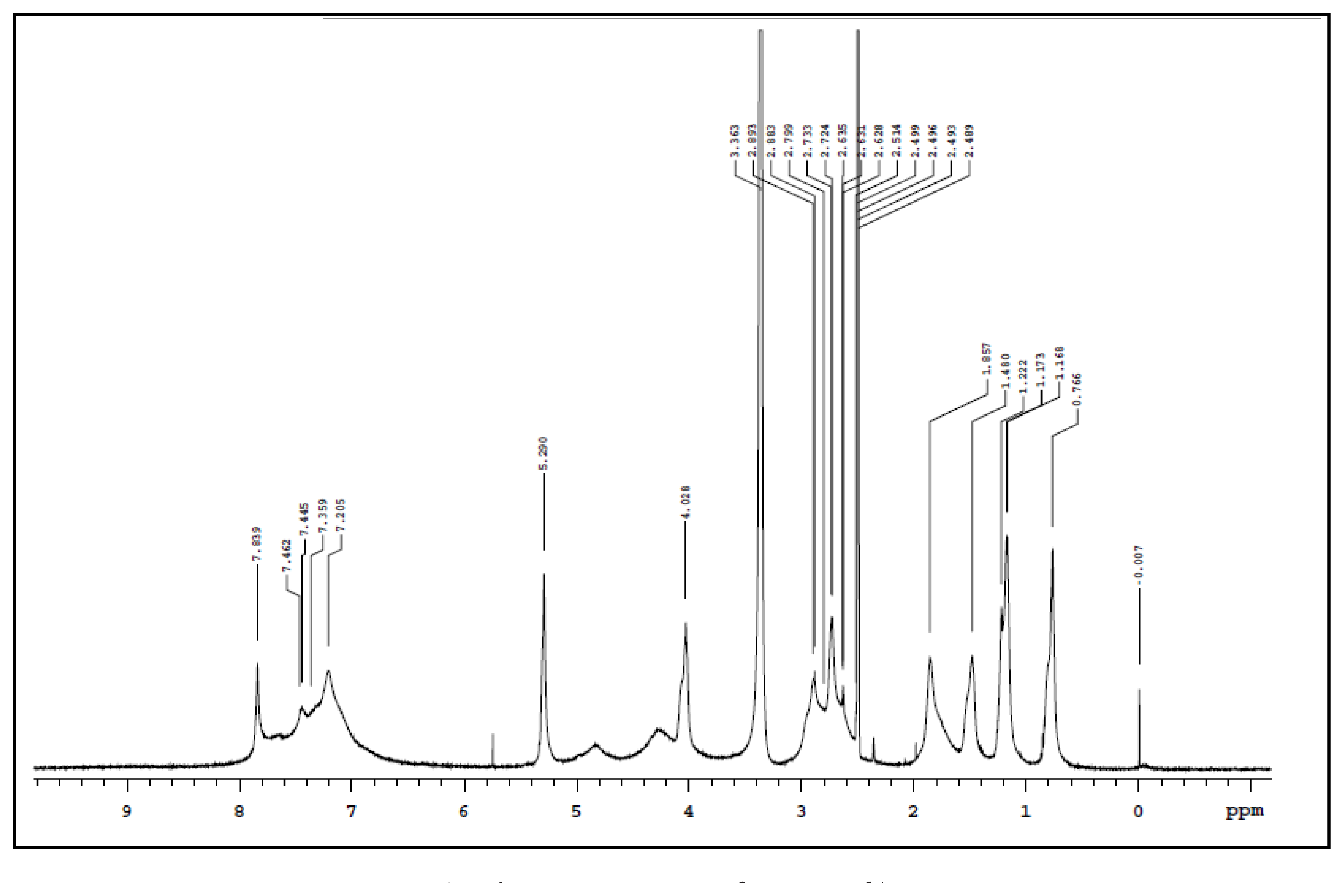

Supplement: Figure S20 — 1H NMR spectrum of compound ZnPc-I. [file tjc-48-06-800s20.tif]

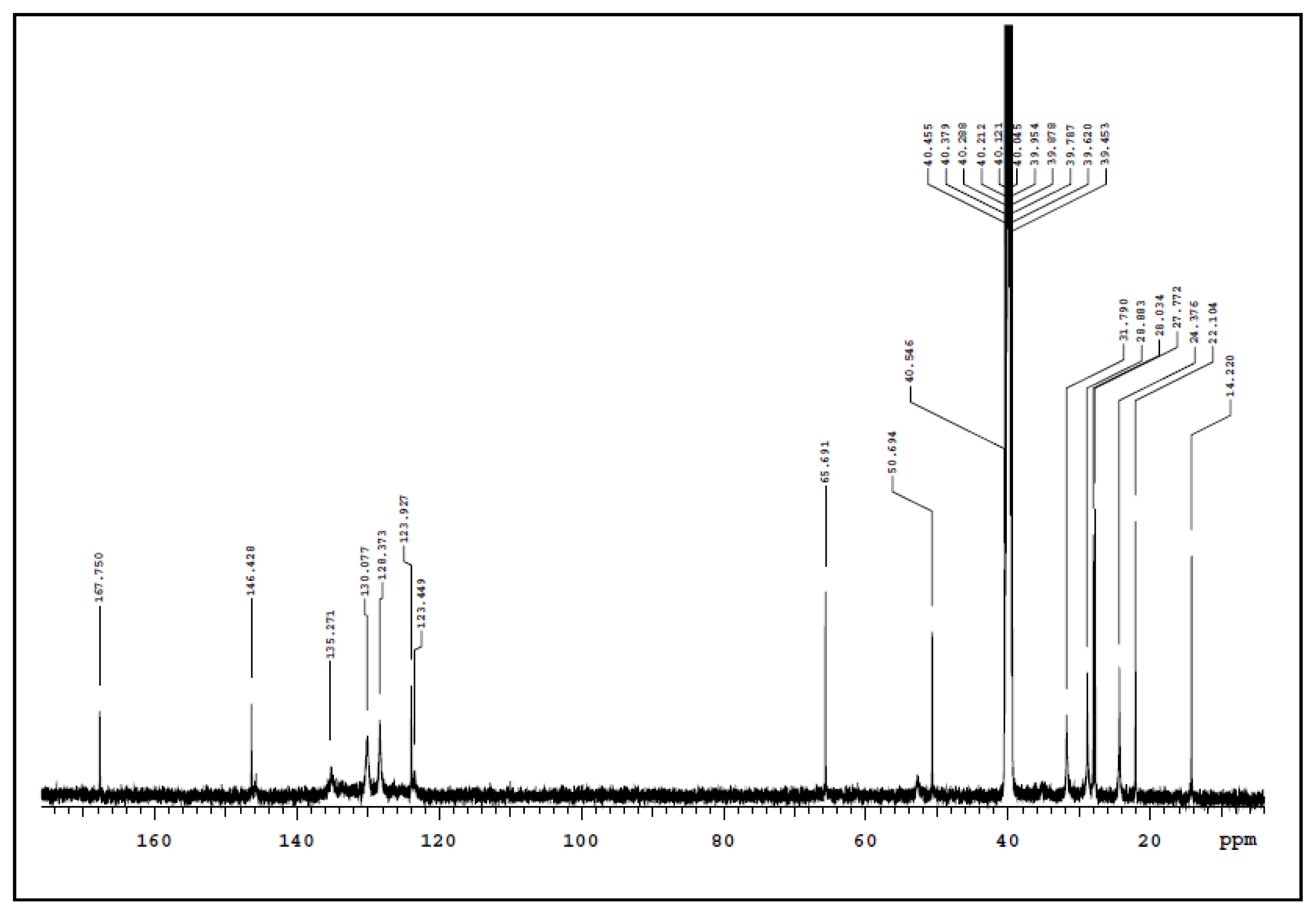

Supplement: Figure S21 — 13C NMR spectrum of compound ZnPc-I. [file tjc-48-06-800s21.tif]

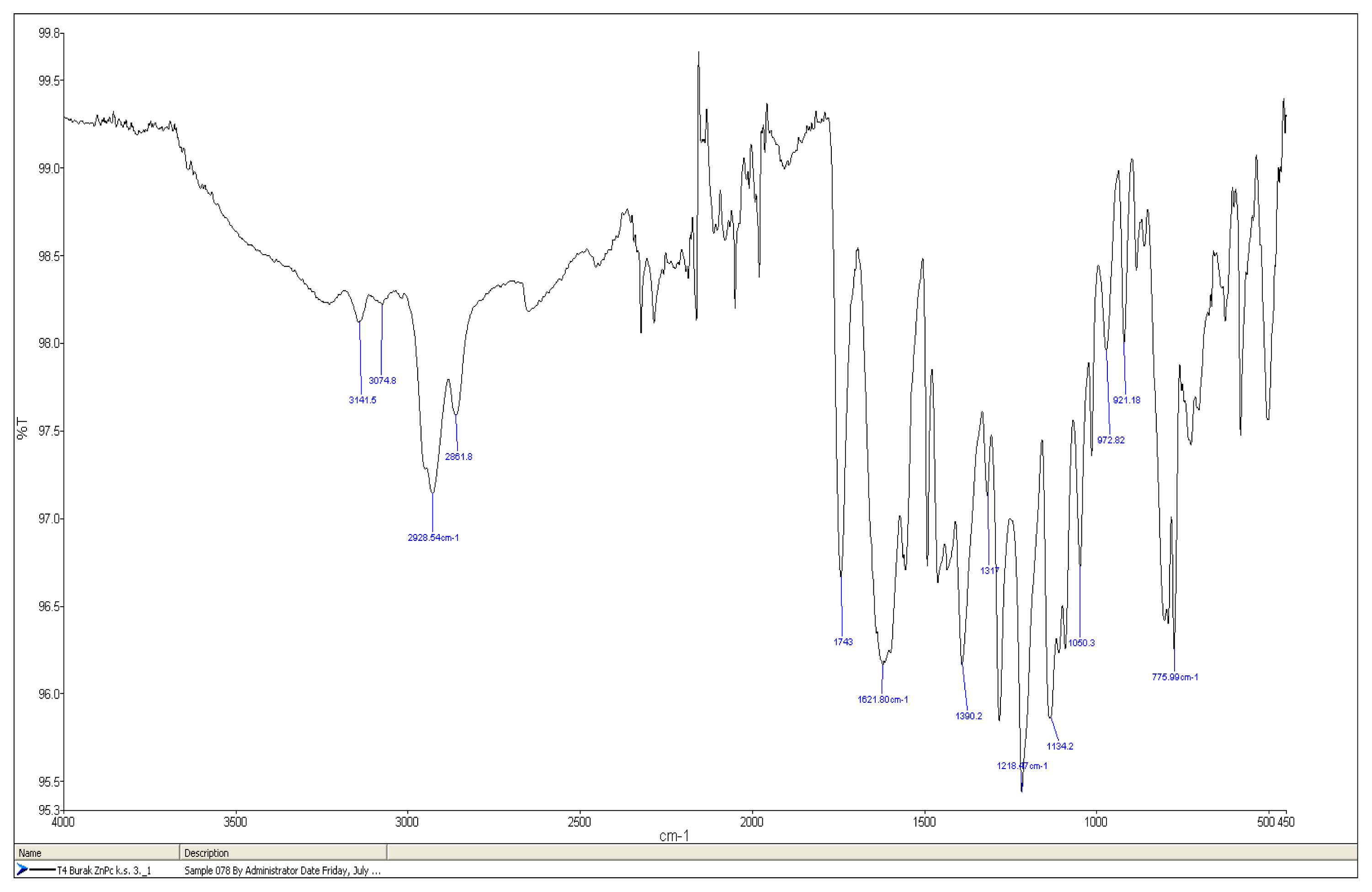

Supplement: Figure S22 — FTIR spectrum of compound ZnPc-I. [file tjc-48-06-800s22.tif]

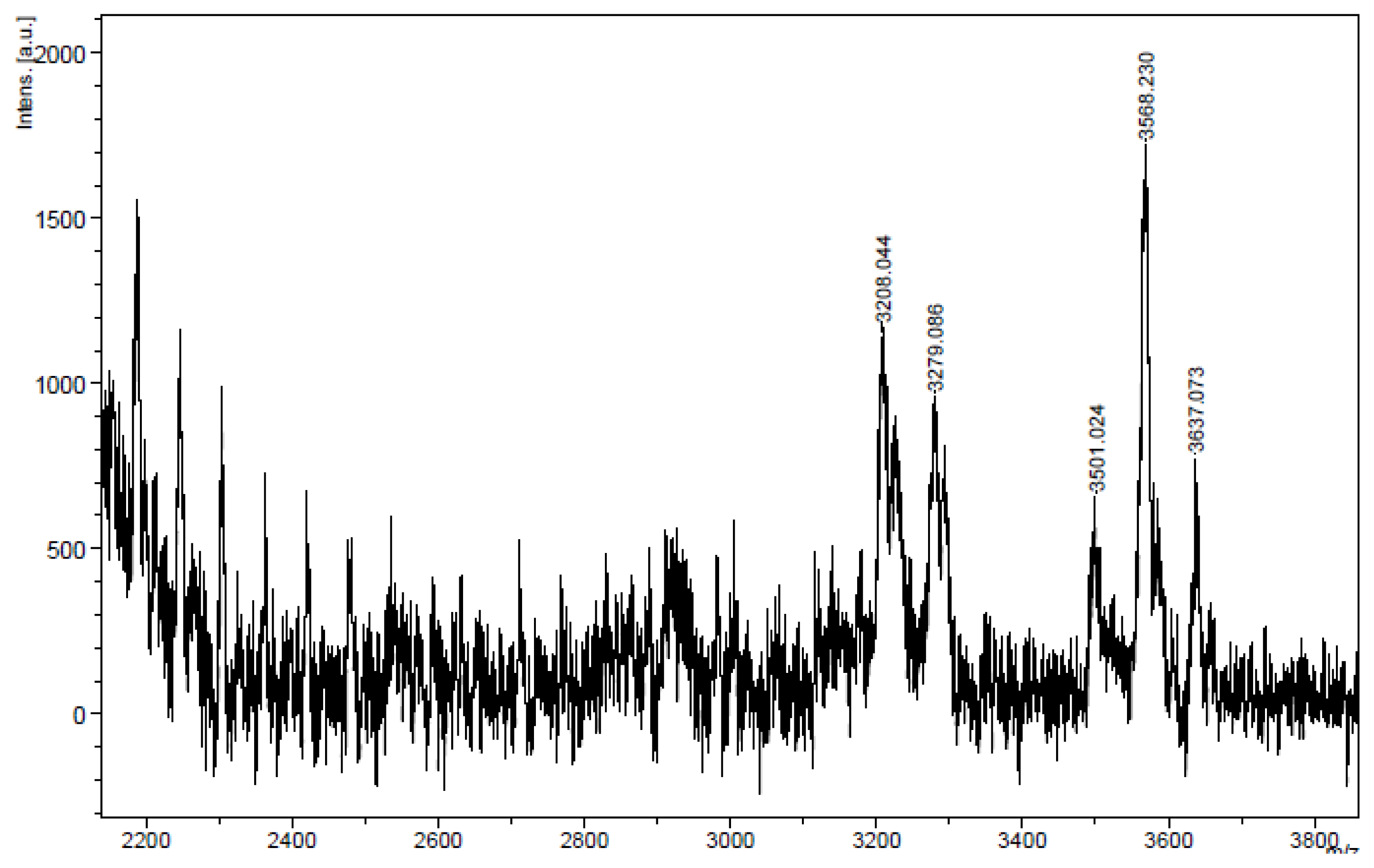

Supplement: Figure S23 — Mass spectrum of compound ZnPc-I. [file tjc-48-06-800s23.tif]

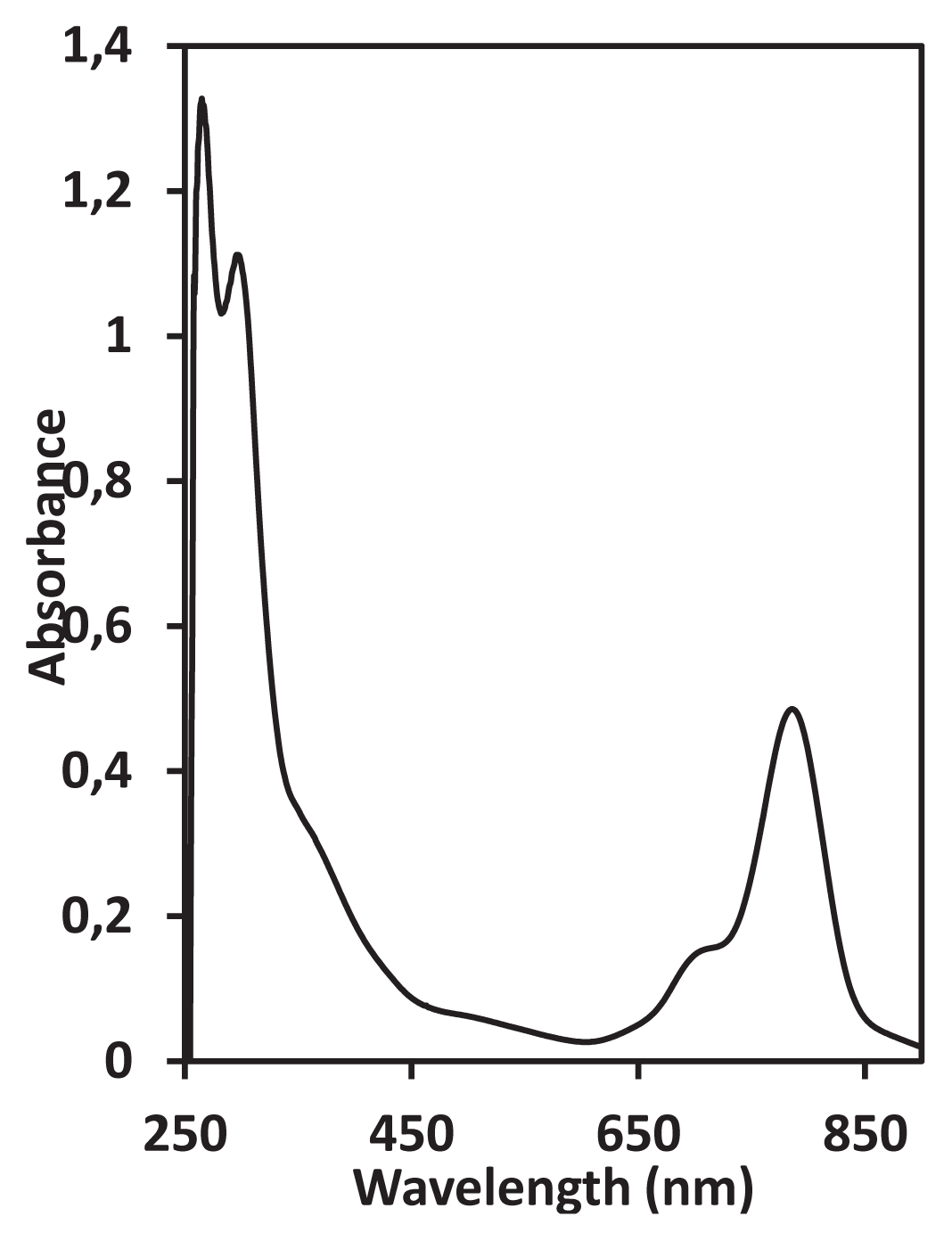

Supplement: Figure S24 — UV-vis spectrum of compound ZnPc-I (1×10^−5^ M in DMSO). [file tjc-48-06-800s24.tif]
